# Boosting Adsorption and Selectivity of Acetylene by Nitro Functionalisation in Copper(II)‐Based Metal–Organic Frameworks

**DOI:** 10.1002/anie.202417183

**Published:** 2024-12-27

**Authors:** Lixia Guo, Xue Han, Jiangnan Li, Weiyao Li, Yinlin Chen, Pascal Manuel, Martin Schröder, Sihai Yang

**Affiliations:** ^1^ College of Chemistry and Molecular Engineering Beijing National Laboratory for Molecular Sciences Peking University Beijing 100871 China; ^2^ Department of Chemistry University of Manchester Manchester M13 9PL UK; ^3^ College of Chemistry Beijing Normal University Beijing 100875 China; ^4^ ISIS Facility Rutherford Appleton Laboratory Chilton OX11 0QX UK

## Abstract

Purification and storage of acetylene (C_2_H_2_) are important to many industrial processes. The exploitation of metal–organic framework (MOF) materials to address the balance between selectivity for C_2_H_2_ *vs* carbon dioxide (CO_2_) against maximising uptake of C_2_H_2_ has attracted much interest. Herein, we report that the synergy between unsaturated Cu(II) sites and functional groups, fluoro (−F), methyl (−CH_3_), nitro (−NO_2_) in a series of isostructural MOF materials MFM‐190(R) that show exceptional adsorption and selectivity of C_2_H_2_. At 298 K, MFM‐190(NO_2_) exhibits an C_2_H_2_ uptake of 216 cm^3^ g^−1^ (320 cm^3^ g^−1^ at 273 K) at 1.0 bar and a high selectivity for C_2_H_2_/CO_2_ (up to ~150 for v/v = 2/1) relevant to that in the industrial cracking stream. Dynamic breakthrough studies validate and confirm the excellent separation of C_2_H_2_/CO_2_ by MFM‐190(NO_2_) under ambient conditions. In situ neutron powder diffraction reveals the cooperative binding, packing and selectivity of C_2_H_2_ by unsaturated Cu(II) sites and free −NO_2_ groups.

## Graphical abstract

The isostructural MOF materials, MFM‐190(R) (R=−F, −CH_3_, −NO_2_) exhibit exceptional acetylene (C_2_H_2_) adsorption and selectivity. MFM‐190(NO_2_) shows a high C_2_H_2_ uptake of 216 cm^3^ g^−1^ at 1 bar, 298 K and excellent C_2_H_2_/CO_2_ selectivity (up to ~150 for v/v = 2/1). Dynamic breakthrough experiments confirm efficient separation under ambient conditions, supported by in situ neutron powder diffraction studies revealing cooperative binding of C_2_H_2_ at unsaturated Cu(II) and −NO_2_ sites.

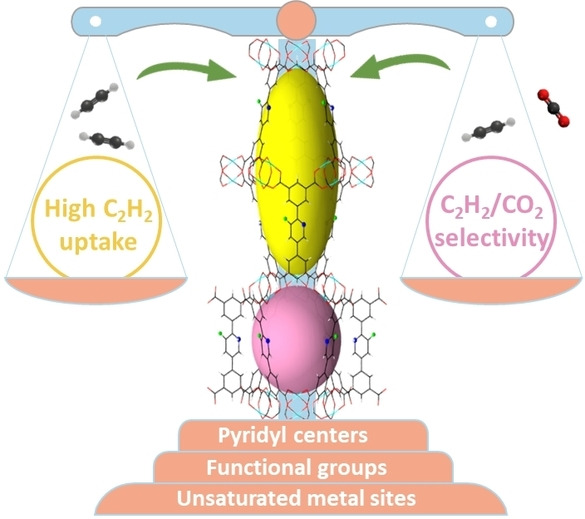

Acetylene (C_2_H_2_) is a crucial precursor for the manufacturing of many chemicals and materials, such as vinyl chloride, synthetic rubber and polyester plastics.[^1^, ^2^, ^3^, ^4^] State‐of‐the‐art production of C_2_H_2_ involves the partial combustion of natural gas (>80 % methane) or hydrocarbon cracking, where carbon dioxide (CO_2_) is a key byproduct that must be removed to produce high purity C_2_H_2_ for downstream applications.[^5^, ^6^] However, the separation of C_2_H_2_ from CO_2_ is highly challenging due to their similar properties (same kinetic diameter of 3.3 Å; molecular size: 3.2×3.3×5.4 Å for CO_2_ and 3.3×3.3×5.7 Å for C_2_H_2_; boiling point: 194.7 K for CO_2_; 189.3 K for C_2_H_2_).[^7^, ^8^] Current technologies rely on solvent extraction or cryogenic distillation, which are energy‐intensive and associated with safety hazards.^[9]^ In addition, the explosive nature of C_2_H_2_ raises concerns regarding its transportation and storage with the storage pressure being limited to below 2.0 bar.^[10]^ There is, therefore, a critical need to explore new adsorption‐based technologies to enable energy‐efficient purification and safe storage of C_2_H_2_.^[11]^

Metal–organic framework (MOF) materials have been investigated widely for gas adsorption and separation owing to their adjustable pore environment.[^9^, ^10^, ^11^, ^12^] However, the optimisation of selectivity between C_2_H_2_ and CO_2_ against the C_2_H_2_ uptake remains a challenging task.[^13^, ^14^] For example, MOFs with large pores often exhibit high gas uptakes, but with limited selectivity for C_2_H_2_/CO_2_.^[15]^ Conversely, narrow‐pored MOFs can achieve high selectivity at the expense of low C_2_H_2_ uptake.^[16]^ Various strategies have been developed to address this conundrum, including introduction of unsaturated metal sites,^[17]^ regulation of pore size,^[14]^ and ligand functionalisation.^[7]^ Pyridyl functionalised NbO‐type MOFs constructed from [Cu_2_(O_2_CR)_4_] paddlewheels and organic linker are particularly appealing for C_2_H_2_ adsorption because the N‐donors in the pyridyl groups can bind C_2_H_2_ via H−C≡C−H⋅⋅⋅N hydrogen bonding, thus improving C_2_H_2_ uptake.[^17^, ^18^, ^19^, ^20^, ^21^] For example, ZJU‐5 that incorporates Lewis basic pyridyl sites exhibits a high C_2_H_2_ uptake of 193 cm^3^ g^−1^ at 298 K and 1.0 bar.^[17]^ Additionally, the introduction of functional groups within the pore interior can strengthen the host–guest interactions, thus promoting C_2_H_2_ uptake.^[22]^ However, NbO‐type MOFs have been rarely explored for the separation of C_2_H_2_/CO_2_ owing to their high porosity and thus poor selectivities.

Herein, we report a strategy to develop and utilise the synergy between unsaturated Cu(II) sites, pyridyl centres and pendant functional groups in a series of NbO‐type MOFs, denoted as MFM‐190(R) (R=−F, −CH_3_, −NO_2_), to boost the adsorption of C_2_H_2_ while simultaneously promoting the separation of C_2_H_2_/CO_2_. At 273 K and 1.0 bar, MFM‐190(NO_2_) exhibits an exceptional C_2_H_2_ uptake capacity of 320 cm^3^ g^−1^, surpassing that of MFM‐190(CH_3_) (300 cm^3^ g^−1^) and MFM‐190(F) (282 cm^3^ g^−1^). More importantly, at 298 K and 1.0 bar, MFM‐190(NO_2_) demonstrates simultaneously high C_2_H_2_ uptake (216 cm^3^ g^−1^) and a high selectivity for C_2_H_2_ *vs* CO_2_ (at v/v = 2/1, relevant to industrial cracking stream) of up to ~150 based on analysis using ideal adsorbed solution theory (IAST). This compares favourably with state‐of‐the‐art materials. Furthermore, breakthrough experiments show excellent separation performance for MFM‐190(NO_2_), affording clear separation of C_2_H_2_/CO_2_, while cycling breakthrough experiments confirm the stability and recyclability of MFM‐190(NO_2_). Direct visualisation of binding of C_2_H_2_ and CO_2_ molecules within the pores of MFM‐190(NO_2_) has been achieved using in situ neutron powder diffraction (NPD) analysis, which affords important insights into the high adsorption and selectivity of C_2_H_2_ at a molecular level.

Isostructural MFM‐190(R) (R=−F, −CH_3_, −NO_2_) were obtained by introducing −F, −CH_3_, and −NO_2_ groups onto the parent ligand used for the preparation of MFM‐190(H) (Figure S1).^23^ Powder X‐ray diffraction (PXRD) analysis confirmed their phase purity (Figure S2), and their crystal structure derived from NPD data shows two types of metal‐ligand cages, which are alternately stacked in a 1 : 1 ratio to afford an open NbO‐type structure. The cylindrical cage (size of ca. 18.6×27.4 Å) is encapsulated by twelve [Cu_2_(O_2_CR)_4_] moieties and six organic ligands. The spherical cage (size of 14.7×14.7 Å) is surrounded by six [Cu_2_(O_2_CR)_4_] paddlewheels and twelve organic ligands (Figure 1). The interior walls of these cages, upon de‐solvation, are decorated with unsaturated Cu(II) sites, pyridyl centres as well as pendant functional groups, which can promote the efficient binding and packing of C_2_H_2_ molecules by facilitating their close contacts. MFM‐190(F), MFM‐190(CH_3_) and MFM‐190(NO_2_) show Brunauer–Emmett–Teller (BET) surface areas of 2530, 2550 and 2300 m^2^ g^−1^, respectively, as determined by N_2_ isotherms at 77 K (Figure S3).

**Figure 1:**
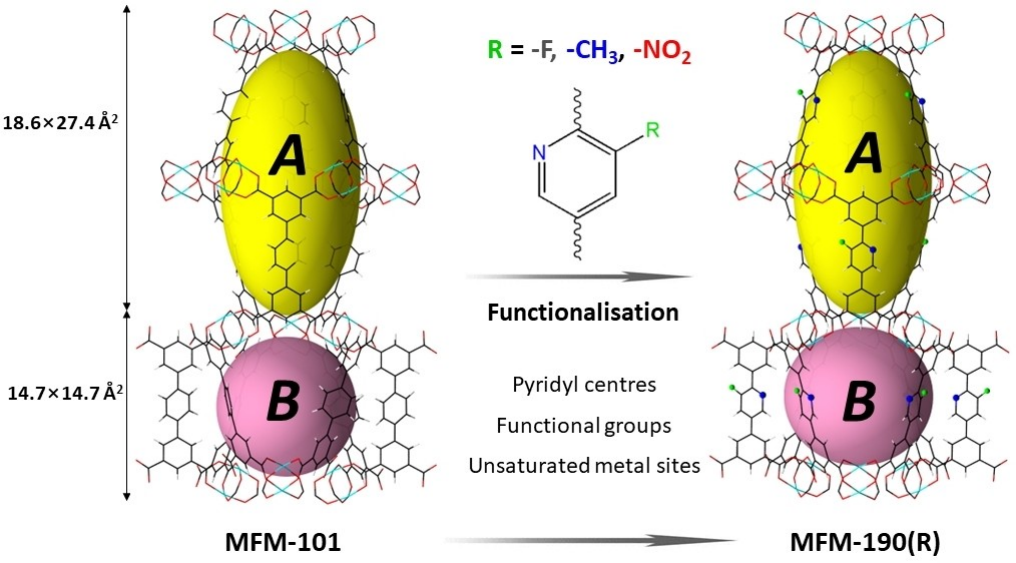
Views of the crystal structures MFM‐101 and MFM‐190(R) derived from the NPD studies (Cu, cyan; C, black; O, red; N, blue; H, grey; R, green; R represents −F, −CH_3_, and −NO_2_). Two types of metal‐ligand cages are interconnected by sharing three [Cu_2_(O_2_CR)_4_] paddlewheel units and three iso‐phthalate moieties.

Adsorption and desorption isotherms of C_2_H_2_ and CO_2_ for MFM‐190(R) were recorded at 273–298 K (Figure 2a–b and Figure S4–S6). Of these three materials, MFM‐190(NO_2_) shows the highest C_2_H_2_ uptake of 320 and 216 cm^3^ g^−1^ at 273 and 298 K, respectively, compared with MFM‐190(CH_3_) (300 and 194 cm^3^ g^−1^) and MFM‐190(F) (282 and 174 cm^3^ g^−1^) (Figure 2a and Figure S7). MFM‐190(NO_2_) also demonstrates an excellent cyclability for C_2_H_2_ as confirmed by pressure‐swing adsorption and desorption (0–500 mbar) at 298 K (Figure S8). The incorporation of relatively bulker functional group (−NO_2_) leads to the reduced pore sizes and surface area compared with −CH_3_ and −F groups, which can facilitate enhanced interaction between C_2_H_2_ and the MOF. By comparison, at 298 K and 1.0 bar, the isostructural pyridyl‐free ZJNU‐36(NO_2_)^[22]^ and −NO_2_‐free ZJU‐5^[15]^ show lower C_2_H_2_ uptakes of 176 and 193 cm^3^ g^−1^, respectively, suggesting the positive impacts of these functional groups on C_2_H_2_ uptakes. Notably, the C_2_H_2_ uptake of MFM‐190(NO_2_) compares favourably with leading MOFs (Table S2).[^1^, ^2^, ^7^, ^9^, ^19^, ^21^, ^22^, ^24^, ^25^, ^26^, ^27^, ^28^, ^29^, ^30^, ^31^, ^32^] Using the crystal density (0.92 g cm^−3^) of MFM‐190(NO_2_), the C_2_H_2_ packing density in the pores is calculated to be 230 g L^−1^ at 298 K and 1.0 bar, which is 196 times that of gaseous C_2_H_2_ (1.17 g L^−1^) and surpasses that of ZJNU‐47 (170 g L^−1^ at 295 K)^[21]^ and NJU‐Bai17 (204 g L^−1^ at 296 K),^[19]^ and is slightly lower than FJU‐90a (256 g L^−1^ at 298 K),^[14]^ indicating the highly efficient packing of C_2_H_2_ molecules within MFM‐190(NO_2_). In contrast, the CO_2_ uptakes of MFM‐190(F), MFM‐190(CH_3_), and MFM‐190(NO_2_) are recorded as 82.9, 90.9 and 86.4 cm^3^ g^−1^, respectively, at 1.0 bar and 298 K (Figure 2b **)**. Interestingly, at 0.1 bar and 298 K, adsorption of C_2_H_2_ in MFM‐190(NO_2_) displayed a rapid and steep increase up to 87 cm^3^ g^−1^, while that for CO_2_ increases linearly with pressure and exhibits a much lower uptake of 12 cm^3^ g^−1^, demonstrating the great potential for their separation. The IAST selectivity for C_2_H_2_/CO_2_ mixtures (v/v = 1/1, 2/1) was calculated using the single‐component isotherms at 298 K (Figure 2c and Figure S10–S12). Notably, MFM‐190(NO_2_) demonstrates a drastically elevated C_2_H_2_/CO_2_ selectivity of 150 at low pressure for C_2_H_2_/CO_2_ mixtures (at v/v = 2/1), surpassing that of MFM‐190(F) (7.2) and MFM‐190(CH_3_) (20). With the increase of pressure, the selectivity gradually stabilises at 11, 2.7 and 5.1 at 1.0 bar for MFM‐190(NO_2_), MFM‐190(F), and MFM‐190(CH_3_), respectively. The IAST selectivity (150–11) of MFM‐190(NO_2_) compares favourably with that of benchmark MOFs, such as CAU‐10(H) (24.2–4.0),^[12]^ FJI−H8 (10.4–5.4),^[33]^ FJU‐112a (4.2),^[34]^ MIL‐160 (10),^[7]^ ZJU‐40 (17–11.5),^[35]^ and ZJU‐50a (30–12) (Figure 2d and Table S2).^[36]^ It is worth noting that MOF materials exhibiting simultaneously high adsorption and selectivity for C_2_H_2_ are exceedingly rare.

**Figure 2:**
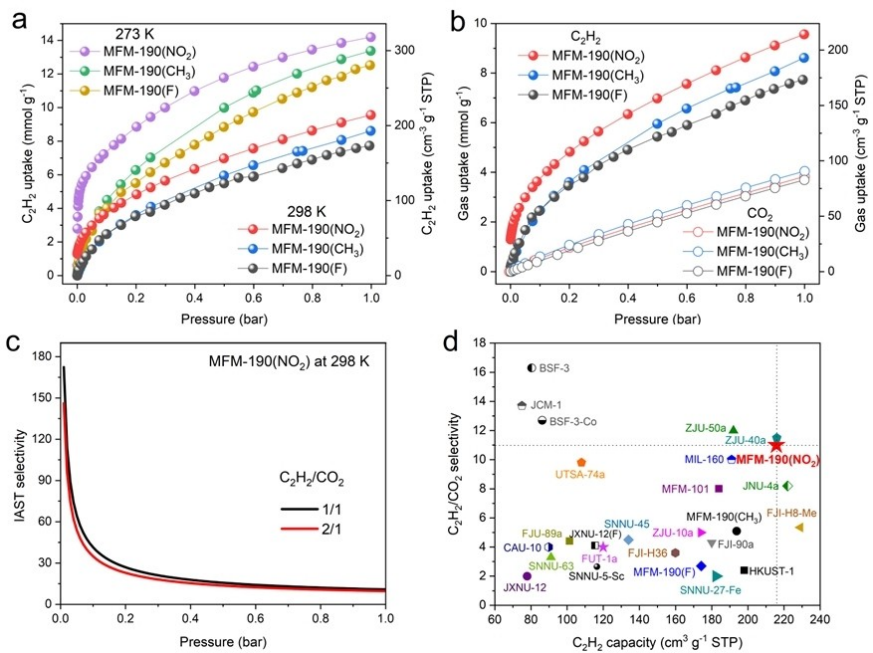
(a) Adsorption isotherms of C_2_H_2_ in MFM‐190(NO_2_), MFM‐190(CH_3_) and MFM‐190(F) at 273–298 K. (b) Adsorption isotherms of C_2_H_2_ and CO_2_ in MFM‐190(NO_2_), MFM‐190(CH_3_) and MFM‐190(F) at 298 K. Desorption isotherms are omitted for clarity and can be found in the Supporting Information . (c) IAST selectivities of C_2_H_2_/CO_2_ (v/v = 1/1, 1/2) of MFM‐190(NO_2_) at 298 K. (d) Comparison of state‐of‐art sorbents for C_2_H_2_ capacity and C_2_H_2_/CO_2_ selectivity at 298 K and 1.0 bar.

We investigated the adsorption kinetic profiles of MFM‐190(NO_2_) at 298 K (Figure S13). MFM‐190(NO_2_) shows rapid diffusion for both C_2_H_2_ and CO_2_, reaching adsorption equilibrium within approximately 6 minutes reflecting the large pores in this material. The binding affinity of MFM‐190(R) toward C_2_H_2_ and CO_2_ was further elucidated by measurement of the isosteric heats of adsorption (*Q*_st_) (Figure S14–S19). Notably, MFM‐190(NO_2_) exhibits an exceptional *Q*_st_ value for C_2_H_2_ (90 kJ mol^−1^) at low surface coverage, significantly higher than that of MFM‐190(F) (37 kJ mol^−1^) and MFM‐190(CH_3_) (38 kJ mol^−1^). The values of *Q*_st_ for CO_2_ for MFM‐190(F) and MFM‐190(CH_3_) are similar, and are slightly lower than that of MFM‐190(NO_2_). The *Q*_st_ value for C_2_H_2_ in MFM‐190(NO_2_) is comparable with MOFs incorporating high‐density unsaturated metal sites, such as Cu^I^@UiO‐66‐(COOH)_2_ (74.5 kJ mol^−1^),^[1]^ NKMOF−Ni (60.3 kJ mol^−1^),^[37]^ ZJU‐74a (45 kJ mol^−1^),^[38]^ and ATC−Cu (79.1 kJ mol^−1^),^[16]^ indicating the critical role of Cu(II) sites and functional groups (−NO_2_ and −N) in binding C_2_H_2_ molecules in MFM‐190(NO_2_). More importantly, the substantial difference in *Q*_st_ values between C_2_H_2_ and CO_2_ in MFM‐190(NO_2_) is consistent with the steep isotherms of C_2_H_2_ at low pressure.

The high static C_2_H_2_ adsorption and C_2_H_2_/CO_2_ selectivity of MFM‐190(NO_2_) motivated us to evaluate its separation performance under dynamic conditions. Initially, single‐component breakthrough experiments of CO_2_ and C_2_H_2_ were conducted using a diluted He flow with a total flow rate of 20 mL min^−1^ through a fixed‐bed packed of activated MFM‐190(NO_2_) at 298 K (Figure S20–S21). The dynamic adsorption capacities for C_2_H_2_ and CO_2_ were calculated to be 3.6 and 0.4 mmol g^−1^, respectively. These values are slightly lower than the static capacities obtained from the isotherms at 298 K at 0.1 bar (4.1 and 0.5 mmol g^−1^, respectively), and such discrepancy is consistent with literature reports.^[39]^ Dynamic breakthrough experiments were performed using mixtures of C_2_H_2_/CO_2_ (v/v = 2/1, 1/1) at 298 K and 1.0 bar (Figure 3a). Clear separation of the equimolar mixture of C_2_H_2_/CO_2_ was achieved, with CO_2_ eluting first at 10 min g^−1^, while no C_2_H_2_ was detected until breakthrough at 55 min g^−1^. The separation factor for MFM‐190(NO_2_) was calculated as 5.5, which is higher than the top‐performing MOF materials, such as JCM‐1 (4.4),^[40]^ ZJU‐50a (4.2),^[36]^ Cu^I^@UiO‐66‐(COOH)_2_ (3.4),^[1]^ CuZn_3_(PDDA)_3_(OH) (3.3),^[41]^ but lower than that of BSF‐3 (16.3)^[15]^ and JNU‐4a (12.8).^[9]^ Importantly, MFM‐190(NO_2_) displays simultaneously high C_2_H_2_ uptake (216 cm^3^ g^−1^) and high separation factor (5.5), surpassing those of benchmark MOFs, such as SNNU‐27‐Fe (182 cm^3^ g^−1^, 2.8),^[27]^ MIL‐160 (191 cm^3^ g^−1^, 1.7),^[7]^ Cu^I^@UiO‐66‐(COOH)_2_ (48 cm^3^ g^−1^ and 3.4).^[1]^ High‐purity C_2_H_2_ (>99.9 %) has been harvested from MFM‐190(NO_2_), resulting in a productivity of 4.0 mol kg^−1^ at 298 K, which is also higher than those of benchmark materials, such as ZJU‐74a (3.6 mol kg^−1^),^[38]^ Cu^I^@UiO‐66‐(COOH)_2_ (2.9 mol kg^−1^),^[1]^ and CAU‐10 (3.3 mol kg^−1^).^[3]^ Moreover, MFM‐190(NO_2_) shows excellent separation of a mixture of C_2_H_2_/CO_2_ (v/v = 2/1), which is relevant to separations for industrial cracking streams (Figure 3b).^[15]^ When purging with He at 298 K, the fixed‐bed can be regenerated completely, and the recyclability of the medium for separation of C_2_H_2_/CO_2_ (v/v = 2/1) was confirmed for three cycles with excellent stability (Figure 3c).

**Figure 3:**
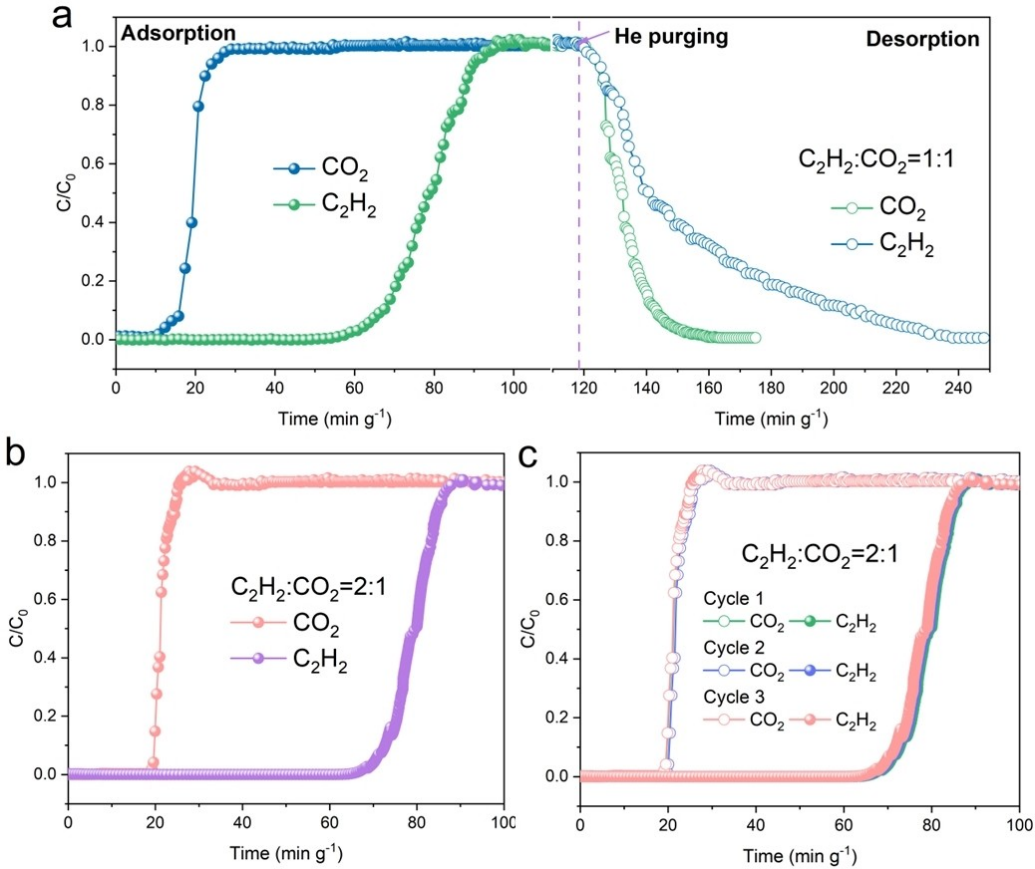
(a, b) Breakthrough curves for mixtures of C_2_H_2_/CO_2_ (a) v/v = 1/1 and (b) 2/1 diluted in He at a flow rate of 20 mL min^−1^ at 298 K and 1.0 bar over a fixed‐bed of MFM‐190(NO_2_) (sample weight: 0.55 g) and regeneration of sorbent. (c) The recyclability of MFM‐190(NO_2_) for the separation of C_2_H_2_/CO_2_ (v/v = 2/1) over three cycles (the saturated sorbent was regenerated by heating at 373 K under a flow of He for 30 mins between cycles).

In situ NPD analysis was performed to determine the preferred binding domains for adsorbed gas (C_2_D_2_, CO_2_) molecules within MFM‐190(NO_2_) (Figure S22–S24 and Table S3–S8). Rietveld refinements revealed seven distinct binding sites for C_2_D_2_ (Figure 4) and four sites for CO_2_ (Figure 5). In the structure of MFM‐190(NO_2_)⋅(C_2_D_2_)_5.2_, site I (C_2_D_2_/Cu=0.359) is within the spherical cage ***B*** and exhibits strong binding interactions with the unsaturated Cu(II) sites [Cu⋅⋅⋅C≡C_C2D2_=3.05(2) Å]. In addition, site I is stabilised by intermolecular interactions with site II and site IV [C_I_⋅⋅⋅C_II_=3.39(1) Å and C_I_⋅⋅⋅C_IV_=2.55(1) Å]. Site II (C_2_D_2_/Cu=0.192) is situated within both cages and binds to −NO_2_ groups and phenyl rings via electrostatic interactions [D_C2D2_⋅⋅⋅O_NO2_=2.54(1) Å; D_C2D2_⋅⋅phenyl rings=2.81(3) Å]. Sites III and IV are located only in cage ***B*** where C_2_D_2_ molecules bind to unsaturated Cu(II) sites [Cu⋅⋅⋅D_C2D2_=2.75(4) Å; Cu⋅⋅⋅C_C2D2_=3.35(1) Å]. Sites V–VII are found in the cylindrical cage ***A*** and stabilised by intermolecular interactions [C_V_⋅⋅⋅C_VI_=2.68(2) Å and by relatively weak electrostatic interactions with pyridyl sites [D_C2D2_⋅⋅⋅pyridyl sites=2.99(1) Å] and phenyl rings [C_VII_⋅⋅⋅phenyl rings=3.79(2) Å and 3.96(1) Å]. Notably, the smaller spherical cage ***B*** plays an important role in C_2_D_2_ adsorption, and intermolecular interactions between each site stabilises further the packing of C_2_D_2_ molecules to give an enhanced overall uptake (Figure 4c).

**Figure 4:**
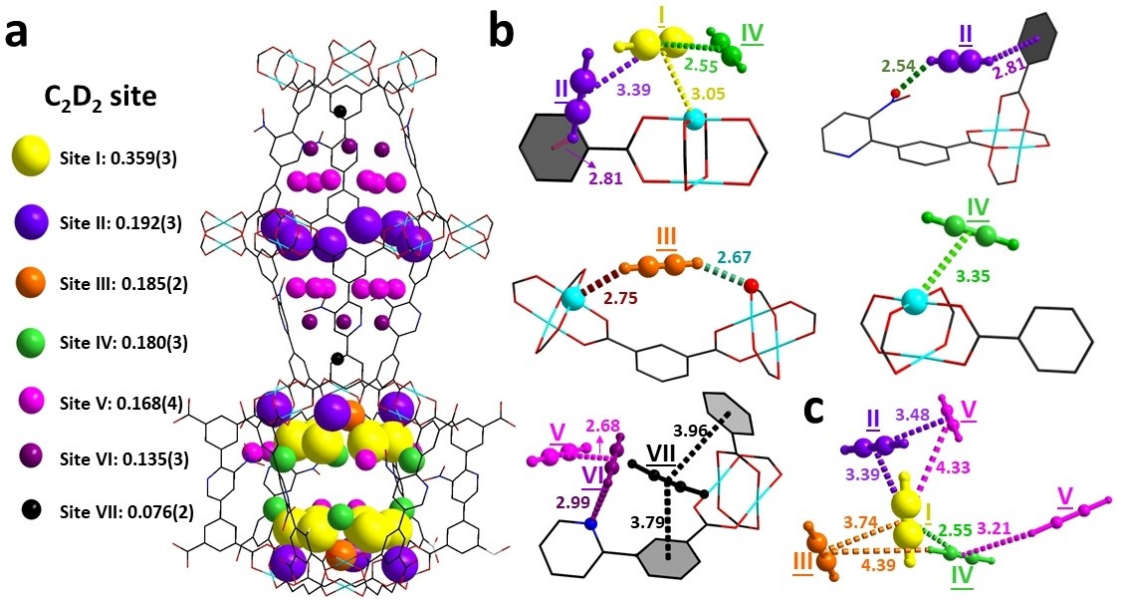
Views of host–guest interactions in C_2_D_2_‐loaded MFM‐190(NO_2_); all structural models were determined from Rietveld refinements of in situ NPD data collected at 10 K. The interatomic distances are quoted in angstroms (Å). The occupancy of each site has been converted into C_2_D_2_ per Cu for clarity. The radii of the coloured spheres are proportional to the corresponding crystallographic occupancies. Cu, cyan; C, black; O, red; N, blue; H, grey. (a) Views of the distribution of C_2_D_2_ in MFM‐190(NO_2_)⋅(C_2_D_2_)_5.2_. (b) Detailed views of host–guest interactions between MFM‐190(NO_2_) and C_2_D_2_. (c) Views of the packing of adsorbed C_2_D_2_ molecules within cage ***B***. (site I: yellow, site II: purple, site III: orange, site IV: green, site V: pink, site VI: violet, site VII: black)

**Figure 5:**
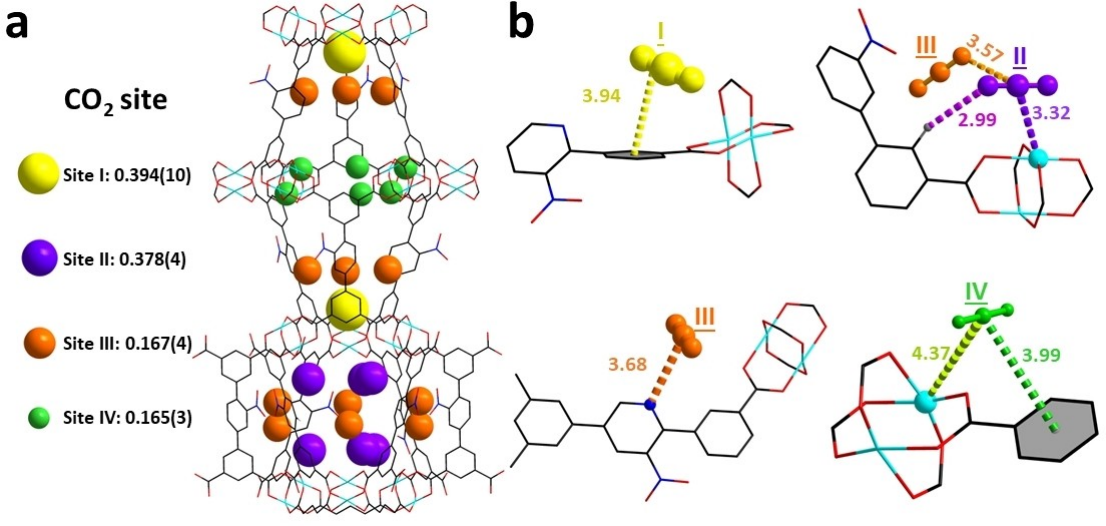
Views of host–guest interactions in CO_2_‐loaded MFM‐190(NO_2_); all structural models were determined from Rietveld refinements of in situ NPD data collected at 10 K. The interatomic distances quoted in angstroms (Å). The occupancy of each site has been converted into CO_2_ per Cu for clarity. The radii of the coloured spheres are proportional to the corresponding crystallographic occupancies. Cu, cyan; C, black; O, red; N, blue; H, grey. (a) Views of the distribution of CO_2_ in MFM‐190(NO_2_)⋅(CO_2_)_3.1_. (b) Detailed views of host–guest interactions between MFM‐190(NO_2_) and CO_2_ (site I: yellow, site II: purple, site III: orange, site IV: green).

For CO_2_‐loaded MFM‐190(NO_2_), perhaps surprisingly, the most favourable adsorption site I (CO_2_/Cu=0.394) was located at the centre of the [Cu_2_(O_2_CR)_4_] paddlewheels, stabilised by electrostatic interactions between phenyl rings and O_CO2_ [phenyl rings⋅⋅⋅O_CO2_=3.94(1) Å]. Site II (CO_2_/Cu=0.378) is situated in the spherical cage ***B*** and binds to the unsaturated Cu(II) site [Cu⋅⋅⋅O_CO2_=3.32(1) Å], and is further stabilised by the hydrogen bonding between H_phenyl rings_ and O_CO2_ [H_phenyl rings_⋅⋅⋅O_CO2_=2.99(1) Å] and intermolecular interactions with site III [O_III_⋅⋅⋅C_II_=3.57(7) Å]. Site III (CO_2_/Cu=0.167) is stabilised by the interactions involving pyridyl sites and CO_2_ [N⋅⋅⋅C_CO2_=3.68(4) Å]. Site IV (CO_2_/Cu=0.165), located at the centre of cylindrical cage ***A***, features C atoms in close proximity to phenyl rings [C_CO2_⋅⋅⋅phenyl rings=3.99(2) Å], and is also stabilised by weak interactions with the Cu(II) site [Cu⋅⋅⋅ C_CO2_=4.37(1) Å]. Thus, the NPD study confirms unequivocally the presence of multiple binding sites and strong affinity for C_2_D_2_ in MFM‐190(NO_2_) compared with CO_2_, directly supporting the observed high adsorption and selectivity of C_2_H_2_.

In summary, we have confirmed that the synergy between unsaturated metal sites, pyridyl groups and pendant functional groups promote the adsorption and selectivity of C_2_H_2_ in NbO‐type MFM‐190(R) (R =−F, −CH_3_, −NO_2_) materials. MFM‐190(NO_2_) shows an exceptional C_2_H_2_/CO_2_ selectivity up to 150 (v/v = 2/1) and high C_2_H_2_ uptake of 216 cm^3^ g^−1^ at 298 K and 1.0 bar. MFM‐190(NO_2_) can harvest a polymer‐grade C_2_H_2_ stream (>99.9 %) with an exceptional productivity of 4.0 mol kg^−1^. Dynamic breakthrough experiments confirmed excellent separation performance for mixtures of C_2_H_2_/CO_2_. The molecular mechanism of host–guest and guest–guest interactions in MFM‐190(NO_2_) has been elucidated by in situ NPD studies as a function of gas loading for both C_2_D_2_ and CO_2_. Overall, the judicious combination of accessible Cu(II) sites, pyridyl groups, functional groups (particularly −NO_2_) and high porosity is key to the development of efficient sorbents for C_2_H_2_.

## Data and code availability

The crystallographic data in this work have been deposited in the Cambridge Crystallographic Data Centre (CCDC). They can be accessed free of charge from https://www.ccdc.cam.ac.uk/structures/. under accession numbers CCDC: 2310235 [bare MFM‐190(NO_2_)], 2310228 [MFM‐190(NO_2_)⋅(C_2_D_2_)_5.2_], and 2310234 [MFM‐190(NO_2_)⋅(CO_2_)_3.1_]. Synthetic procedures, characterisation, and additional analysis of crystal structures and adsorption results have been presented as Supporting Information. Any additional information required in this paper is available from the corresponding authors upon reasonable request.

The authors declare no competing financial interests.

## Twitter account of research group

## Conflict of Interests

The authors declare no conflict of interest.

## Supporting information

As a service to our authors and readers, this journal provides supporting information supplied by the authors. Such materials are peer reviewed and may be re‐organized for online delivery, but are not copy‐edited or typeset. Technical support issues arising from supporting information (other than missing files) should be addressed to the authors.

Supporting Information

Supporting Information

Supporting Information

Supporting Information

## Data Availability

The data that support the findings of this study are available from the corresponding author upon reasonable request.
