# Supplementary material for: Boosting Adsorption and Selectivity of Acetylene by Nitro Functionalisation in Copper(II)‐Based Metal–Organic Frameworks
Source: Angew Chem Int Ed Engl. 2024 Dec 27;64(6):e202417183. doi: 10.1002/anie.202417183 (PMC11795735; doi:10.1002/anie.202417183)
Supplement: Supplementary file 4 — Supporting Information [file ANIE-64-e202417183-s001.pdf]

Angewandte Chemie
Eine Zeitschrift der Gesellschaft Deutscher Chemiker

Supporting Information

Boosting Adsorption and Selectivity of Acetylene by Nitro Functionalisation in Copper(II)-Based Metal–Organic Frameworks

L. Guo, X. Han, J. Li, W. Li, Y. Chen, P. Manuel, M. Schröder, S. Yang**

Supporting Information

Boosting Adsorption and Selectivity of Acetylene by Nitro Functionalisation in Copper(II)-Based Metal-Organic Frameworks

Lixia Guo,[1,2] Xue Han,[3] Jiangnan Li,[1] Weiyao Li,[2] Yinlin Chen,[2] Pascal Manuel,[4] Martin Schröder[2]* and Sihai Yang[1,2]*

1. College of Chemistry and Molecular Engineering, Beijing National Laboratory for Molecular Sciences, Peking University, Beijing, 100871, China.
2. Department of Chemistry, University of Manchester, Manchester, M13 9PL, UK.
3. College of Chemistry, Beijing Normal University, Beijing 100875, China.
4. ISIS Facility, Rutherford Appleton Laboratory, Chilton, OX11 0QX, UK.

Contents

1. Methods and characterisation 2

2. Structures of ligands 5

3. Powder X-ray diffraction 6

4. Gas adsorption isotherms 6

5. Calculation of IAST selectivity 9

6. Adsorption kinetics of C_2H_2 and CO_2 12

7. Analysis and derivation of the isosteric heats of adsorption 13

8. Dynamic breakthrough experiments 16

9. Neutron powder diffraction 17

10. Supplementary tables 20

11. Supplementary references 29

1. Methods and characterisation

The ligands and MOF samples of MFM-190(F), MFM-190(CH_3) and MFM-190(NO_2) were prepared according to our previously published procedures.[1]

Synthesis of MFM-190(F). 5,5'-(3-Fluoropyridine-2,5-diyl)diisophthalic acid (**1**, 20 mg, 0.0487 mmol) and $Cu(NO_3)_2{\cdot}2.5H_2O$ (60 mg, 0.22 mmol) were dissolved in a mixture of dimethylformamide (DMF) (4.0 mL), and EtOH (2.0 mL) to which was added 0.20 mL of 2.0 M HCl solution (diluted from *conc*. HCl with water). The resulting mixture was sealed in a 15 mL pressure tube and heated in an oil bath at 80 °C for 4 days. The resultant deep blue crystals (yield ~40%) were collected and washed with warm DMF three times. Elemental analysis (% calculated and found): C 43.1 and 43.2; H 2.1 and 2.0; F 3.3 and 3.4; N 2.4 and 2.3. The sample was further immersed in an excess of acetone for three days with frequent exchange of solvent.

Synthesis of MFM-190(CH_3). 5,5'-(3-Methylpyridine-2,5-diyl)diisophthalic acid (**2**, 20 mg, 0.0457 mmol) and $Cu(NO_3)_2{\cdot}2.5H_2O$ (60 mg, 0.22 mmol) were dissolved in a mixture of DMF (4.0 mL), and EtOH (2.0 mL) to which was added 0.20 mL of 2.0 M HCl solution (diluted from *conc*. HCl with water). The resulting mixture was sealed in a 15 mL pressure tube and heated in an oil bath at 80 °C for 4 days. The resultant deep blue crystals (yield ~50%) were collected and washed with warm DMF three times. Elemental analysis (% calculated and found): C 45.5 and 45.1; H 2.6 and 2.4; N 2.4 and 2.3. The sample was further immersed in an excess of acetone for three days with frequent exchange of solvent.

Synthesis of MFM-190(NO_2). 5,5'-(3-Nitropyridine-2,5-diyl)diisophthalic acid (**3**, 20 mg, 0.049 mmol) and $Cu(NO_3)_2{\cdot}2.5H_2O$ (60 mg, 0.22 mmol) were dissolved in a mixture of DMF (4.0 mL), and EtOH (2.0 mL) to which was added 0.20 mL of 2.0 M HCl solution (diluted from *conc*. HCl with water). The resulting mixture was sealed in a 15 mL pressure tube and heated in an oil bath at 80 °C for 4 days. The resultant deep blue crystals (yield ~46%) were then washed with warm DMF three times. Elemental analysis (% calculated and found): C 41.3 and 41.1; H 2.0 and 2.0; N 4.6 and 4.3. The sample was further immersed in an excess of acetone for three days with frequent exchange of solvent.

General characterisation. Powder X-ray diffraction (PXRD) patterns were collected on a Philips X'pert X-ray diffractometer (40 kV and 30 mA) using Cu Kα radiation (λ = 1.5406 Å). Permanent porosity was established from nitrogen (N_2) isotherms recorded on a 3-flex (Micrometrics) instrument at 77 K. Ultra-high purity (99.999%), CP grade N_2 was used as supplied by BOC. PXRD patterns (Figure S2) and N_2 adsorption/desorption isotherms (Figure S3) confirm the successful synthesis of these materials.

Gas adsorption isotherms. Gravimetric sorption isotherms (0−1.0 bar) for C_2H_2 and CO_2 were recorded between 273 K and 298 K (temperature-programmed water bath) on a Hiden Isochema Intelligent Gravimetric Analyzer (IGA) system under ultra-high vacuum using a turbo pumping system. In a typical gas adsorption experiment, *ca.* 50 mg of acetone-exchanged MOF sample was loaded into the IGA system and degassed at 383 K under dynamic high vacuum (10^{-6} mbar) for 12 hours to give a fully desolvated sample. Ultra-pure research grade (99.99%) CO_2 was purchased from BOC and used as received. C_2H_2 was purified by dual-stage cold trap systems operated at 195 K (dry ice) and an activated carbon filter before introduction to the IGA system. For MFM-190(NO_2), the isotherms at 308 K were also collected.

Breakthrough experiments. Dynamic breakthrough experiments were conducted on a Hiden Isochema IGA-003 with ABR attachments and a Hiden Analytical mass spectrometer by using a fixed-bed tube packed with 0.55 g of MFM-190(NO_2) powder. The sample was heated at 383 K under a flow of dry He for 12 h for activation, and then cooled to 298 K. Single-component gas breakthrough experiments with an inlet gas flow rate of 2 mL min^{-1} diluted in a flow of He (total flow rate of 20 mL min^{-1}) were measured through a fixed-bed packed with MFM-190(NO_2). For equimolar mixtures of C_2H_2/CO_2, the flow rate of 2.0 mL min^{-1}/2.0 mL min^{-1} diluted in He was applied. Dynamic breakthrough experiments for 2/1 (v/v) mixtures of C_2H_2/CO_2 were conducted at a flow rate of 2 mL min^{-1} or 1 mL min^{-1} diluted in He. All breakthrough experiments were conducted at a total flow of 20 mL min^{-1} at 298 K. The concentration of gas at the outlet was determined by mass spectrometry and compared with the inlet concentration C_0, where $C/C_0 = 1$ indicates complete breakthrough. To determine the dynamic adsorption capacity, the uptake of each component (n_m) was calculated based on the breakthrough curves by the following equation:

$$V_m = \frac{\int_0^t v_{gas\,out}\, d_t - V_{dead}}{W_{MOF}} \qquad (1)$$

$$n_m = \frac{PV_m}{\mathrm{R}T} \qquad (2)$$

where $v_{\text{gas out}}$ is the flow rate of the specific gas (mL min^{-1}); V_{dead} is the dead volume of the system (mL); W represents the mass of sample packed in the breakthrough bed (g); t is the retention time for the specific gas (min); P is atmospheric pressure (kpa); R is gas constant; T is the measurement temperature (K). The C_2H_2 productivity (q_m) was defined by the amount of breakthrough of C_2H_2, calculated by integration of the breakthrough curves over a period t_1 to t_2 during which the gas purity is equal to 99.9%:

$$q_m = \frac{\int_{t1}^{t2} v_{gas\,out}\, d_t - V_{dead}}{W_{MOF}} \qquad (3)$$

where $v_{\text{gas out}}$ is the flow rate of specific gas (mL min^{-1}); V_{dead} is the dead volume of the system (mL); W represents the mass of sample packed in the breakthrough bed (g).

Analysis of isosteric heat of adsorption. The isosteric enthalpies (Q_st) were calculated using the viral method based upon gas adsorption isotherms at four independent temperatures. The data were fitted using equation 4.

$$ln\,P = ln\,N + \frac{1}{\mathrm{T}}\sum_{i=0}^{m} a_i N^i + \sum_{j=0}^{n} b_j N^j \qquad (4)$$

The isosteric enthalpies were then calculated using the following equation 5.

$$Q_{st} = -\mathrm{R}\sum_{i=0}^{m} a_i N^i \quad (5)$$

where P is pressure (mmHg); N is the adsorption capacity (mmol g^{-1}); T is the temperature (K); a_i *and* b_j are virial coefficients, and m and n are coefficients used to describe the isotherms, usually m ≤6 and n ≤3. R is the gas constant.

Calculation of ideal adsorbed solution theory (IAST) selectivity for gas separation. To estimate the selectivity observed for each substrate isotherm data at 298 K were fitted using the dual-site Langmuir-Freundlich (DSLF) model (equation 6).

$$N^{\circ}(f) = \frac{q_1 b_1 P^{v_1}}{1 + b_1 P^{v_1}} + \frac{q_2 b_2 P^{v_2}}{1 + b_2 P^{v_2}} \quad (6)$$

where P is the pressure of the bulk gas at equilibrium with the adsorbed phase, q_i is the maximum adsorption amount, b_i is the the affinity constant and n_i is the deviation from the simple Langmuir equation. Using this fitting, the IAST selectivity can be calculated from equation 7.

$$S = \frac{x_1/y_1}{x_2/y_2} \quad (7)$$

where x_i is the amount of each component adsorbed and y_i is the mole fraction of each component at equilibrium.

***In situ* neutron powder diffraction experiments.** Neutron powder diffraction (NPD) experiments for bare and gas-loaded MFM-190(NO_2) were undertaken on the WISH diffractometer at the ISIS Facility at Rutherford Appleton Laboratory (UK). The instrument has a solid methane moderator providing a high flux of cold neutrons with a large bandwidth, transported to the sample *via* an elliptical guide. The divergence jaws of WISH system allow tuning of the resolution according to the need of the experiment; in this case, it was setup in high resolution mode. The WISH detectors are 1 m long, 8 mm diameter pixelated 3He tubes positioned at 2.2 m from the sample and arranged on a cylindrical locus covering a 2θ scattering angle of 10−170°. To reduce the background from the sample environment, WISH is equipped with an oscillating radial collimator that defines a cylinder of radius of approximately 22 mm diameter at 90 scattering angle. The sample of desolvated MFM-190(NO_2) was loaded into a cylindrical vanadium sample container with an indium vacuum seal connected to a gas handling system. The sample was degassed at 1×10^{-7} mbar and at 383 K for 12 h with He flushing to remove any remaining trace of guest molecules. The sample was dosed with gas molecules using the volumetric method after warmed to room temperature (298 K) to ensure that the gas is well dispersed. A certain amount of gas was dosed into the vanadium holder containing the sample. The ratio of gas to MOF was calculated through the difference of the partial pressure of the gas in the buffer container (500 mL) before and after dosing, based on the equation $PV = n\mathrm{R}T$, where the T is 298 K, R is gas constant, V

is the dead volume of the system (mL). Data collection for desolvated and gas loaded sample was performed while the temperature was controlled using a He cryostat (10 ± 0.2 K).

2. Structures of ligands

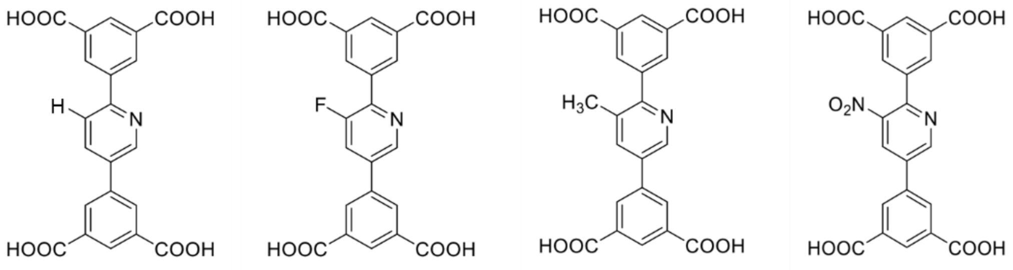

Figure S1. Structures of (**1**) 5,5'-(pyridine-2,5-diyl)diisophthalic acid, (**2**) 5,5'-(3-fluoropyridine-2,5-diyl)diisophthalic acid, (**3**) 5,5'-(3-methylpyridine-2,5-diyl)diisophthalic acid, and (**4**) 5,5'-(3-nitropyridine-2,5-diyl)diisophthalic acid.

3. Powder X-ray Diffraction

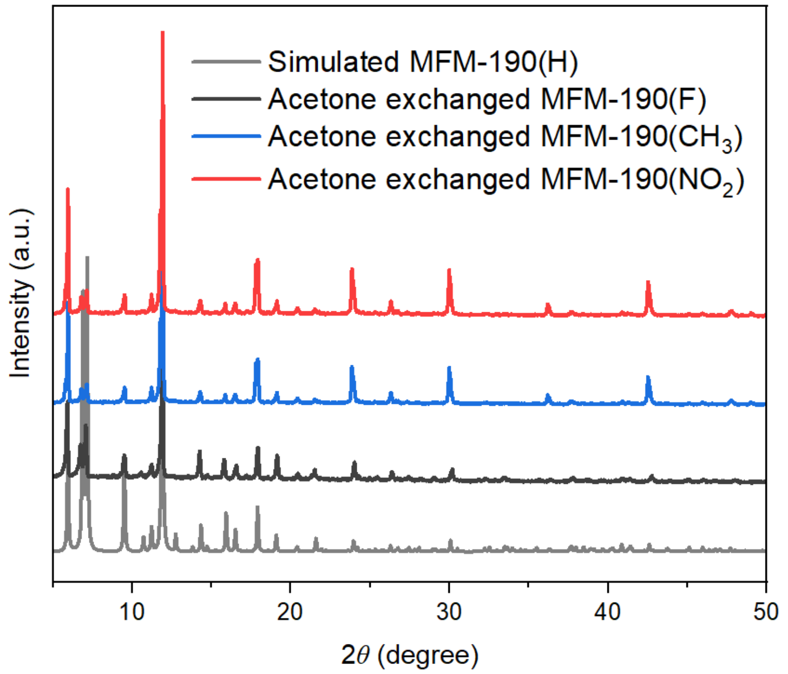

Figure S2. PXRD patterns for simulated MFM-190(H), acetone exchanged MFM-190(F), MFM-190(CH_3) and MFM-190(NO_2).

4. Gas adsorption isotherms

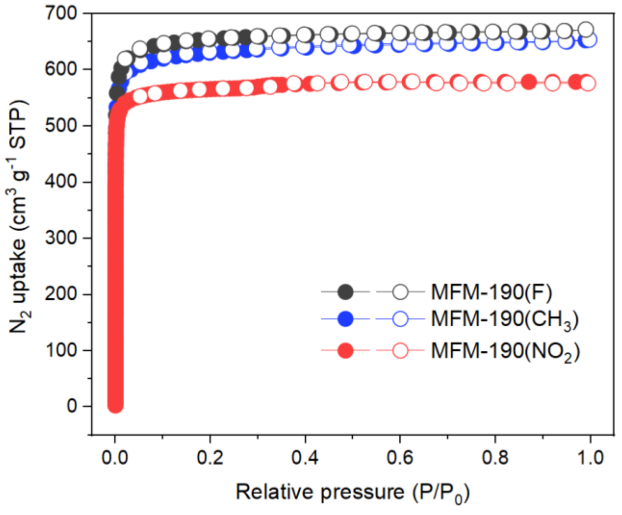

Figure S3. N_2 adsorption/desorption isotherms of desolvated MFM-190(F), MFM-190(CH_3) and MFM-190(NO_2) at 77 K (solid: adsorption; open: desorption).

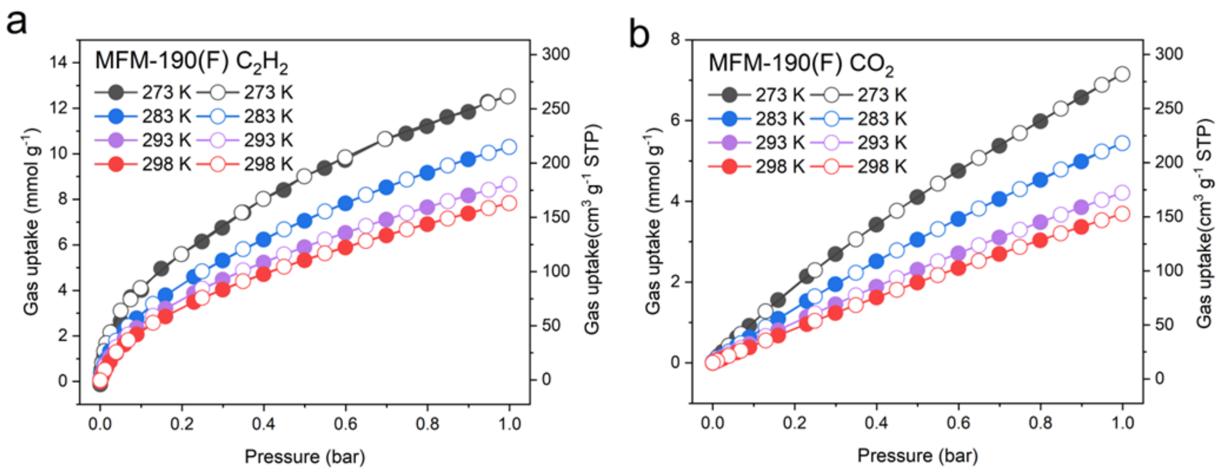

Figure S4. Adsorption/desorption isotherms for (a) C_2H_2 and (b) CO_2 in MFM-190(F) from 273 K to 298 K (solid: adsorption; open: desorption).

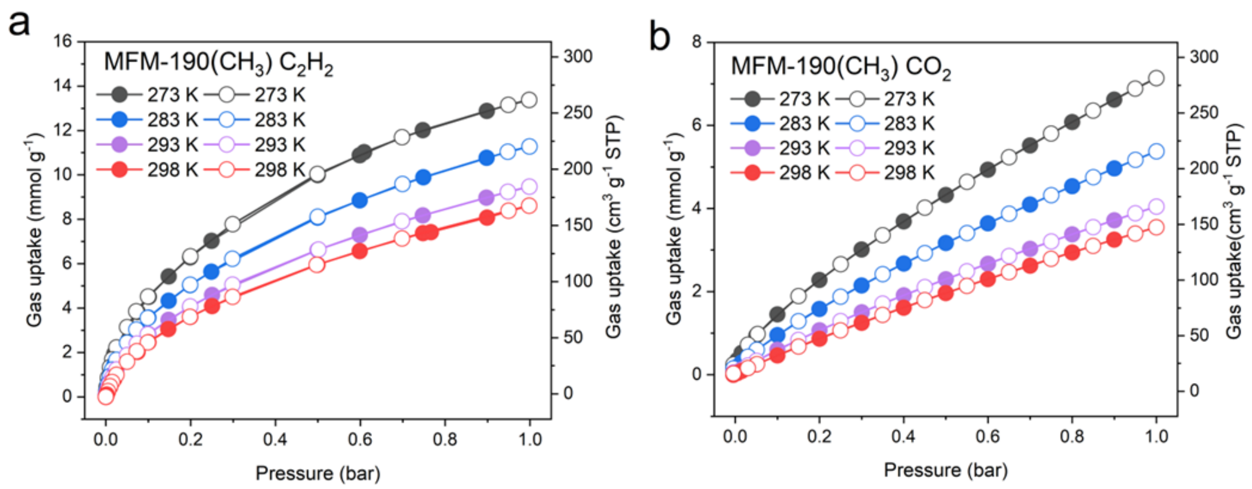

Figure S5. Adsorption/desorption isotherms for (a) C_2H_2 and (b) CO_2 in MFM-190(CH_3) from 273 K to 298 K (solid: adsorption; open: desorption).

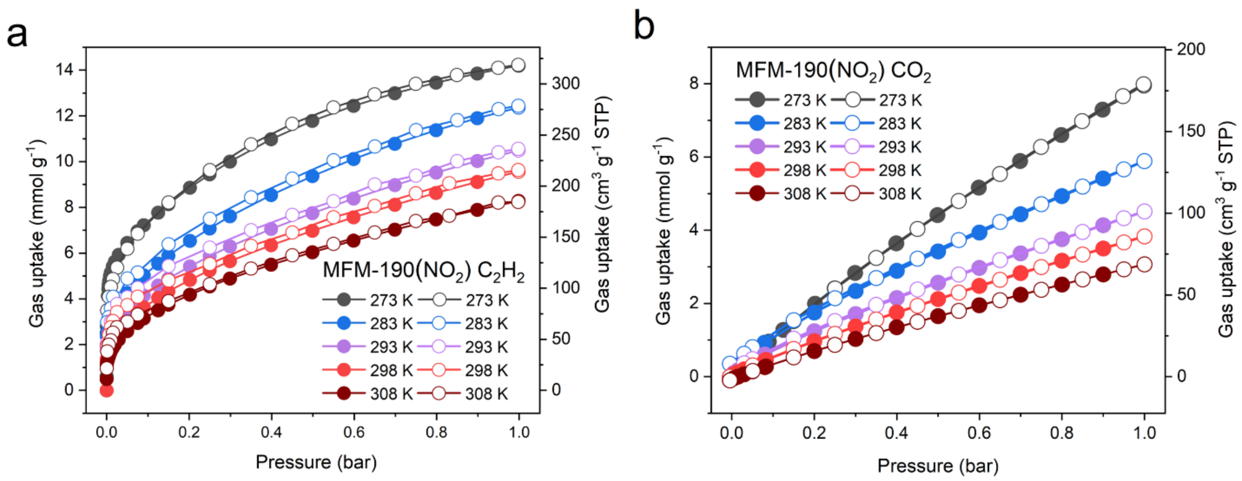

Figure S6. Adsorption/desorption isotherms for (a) C_2H_2 and (b) CO_2 in MFM-190(NO_2) from 273 K to 308 K (solid: adsorption; open: desorption).

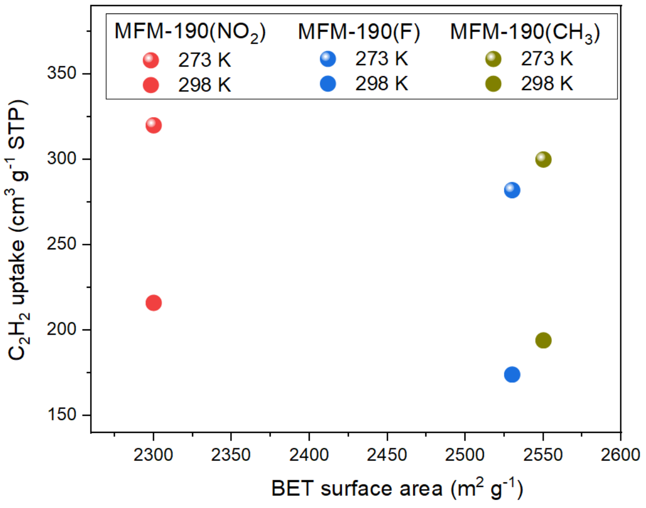

Figure S7. C_2H_2 uptakes (273 K and 298 K, 1.0 bar) *vs* Brunauer-Emmett-Teller (BET) surface area for MFM-190(NO_2), MFM-190(F) and MFM-190(CH_3).

The higher C_2H_2 capacity of MFM-190(NO_2) compared with MFM-190(F) and MFM-190(CH_3) suggests that the surface area is not a direct indicator of C_2H_2 adsorption. Rather, the binding sites within the framework play an important role in determining the overall adsorption capacity.

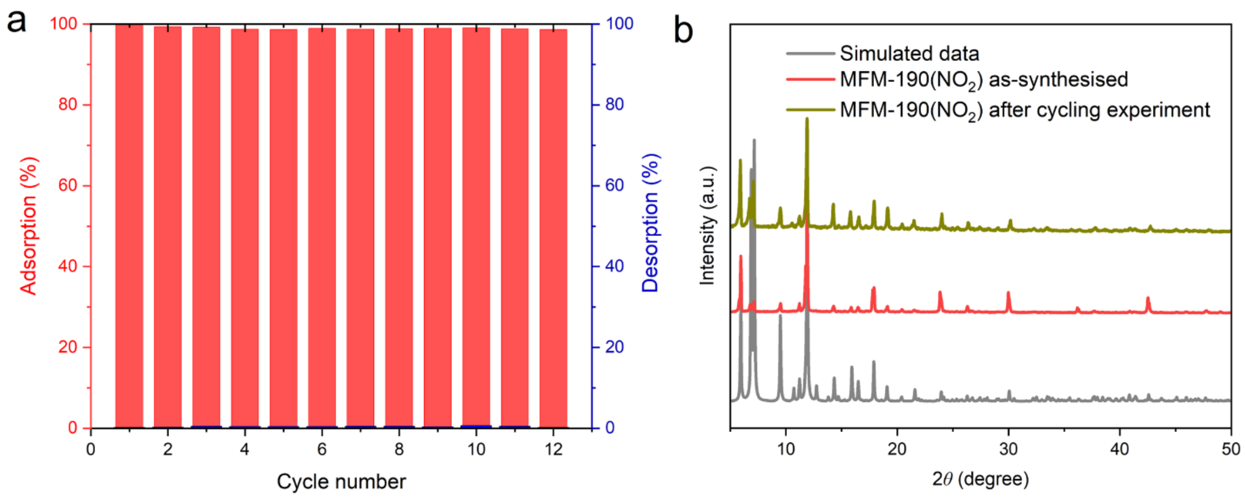

Figure S8. (a) Cycles of pressure-swing adsorption and desorption of C_2H_2 (0–500 mbar) at 298 K in MFM-190(NO_2). (b) PXRD patterns of as-synthesised MFM-190(NO_2) and sample after C_2H_2 cycling experiment.

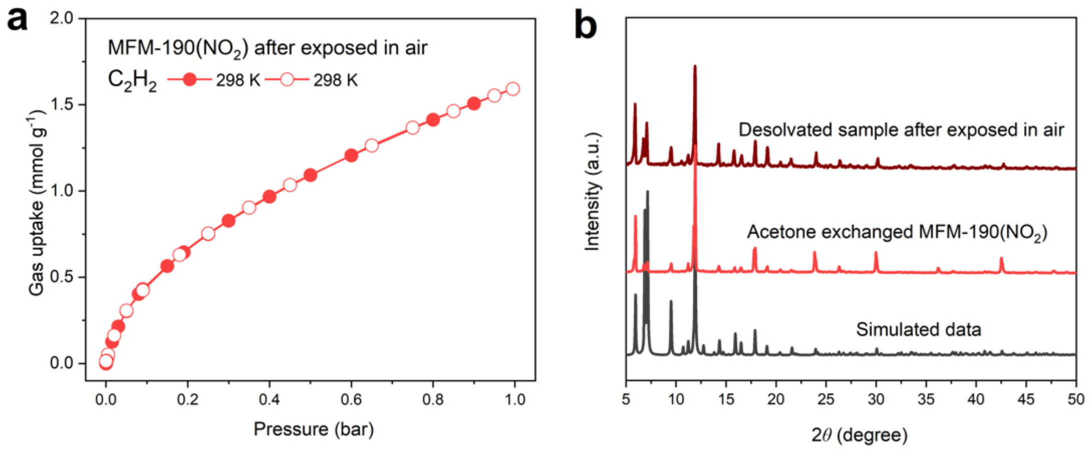

Figure S9. (a) Adsorption/desorption isotherms for C_2H_2 in air-exposed, activated MFM-190(NO_2) at 298 K (solid: adsorption; open: desorption). (b) PXRD pattern of acetone exchanged and air-exposed, activated MFM-190(NO_2).

Following air exposure for 10 h of the activated material, PXRD analysis of MFM-190(NO_2) suggests that the structure remains intact. However, air-exposed MFM-190(NO_2) shows a lower C_2H_2 uptake compared with the pristine sample. This observation underscores the crucial role of the open Cu(II) sites in facilitating high C_2H_2 uptakes, a hypothesis substantiated by the NPD structural analysis.

5. Calculation of IAST selectivity

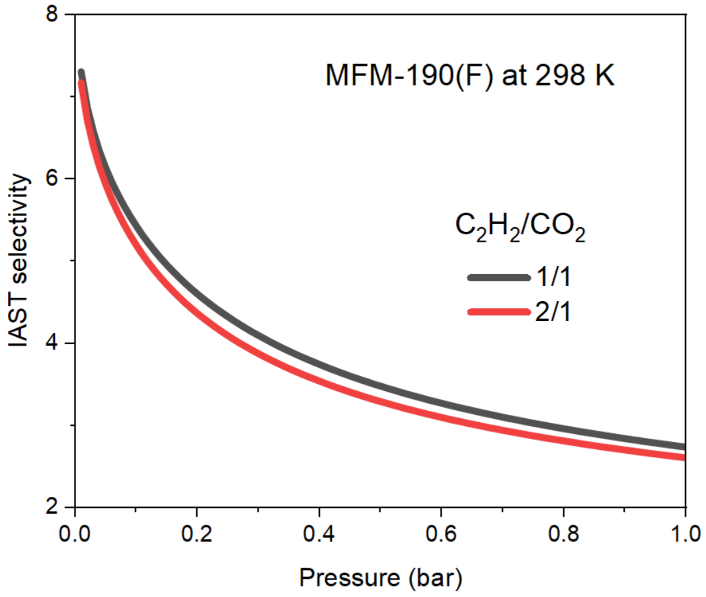

Figure S10. Analysis of IAST selectivities of C_2H_2/CO_2 (v/v = 1/1, 2/1) at 298 K in MFM-190(F).

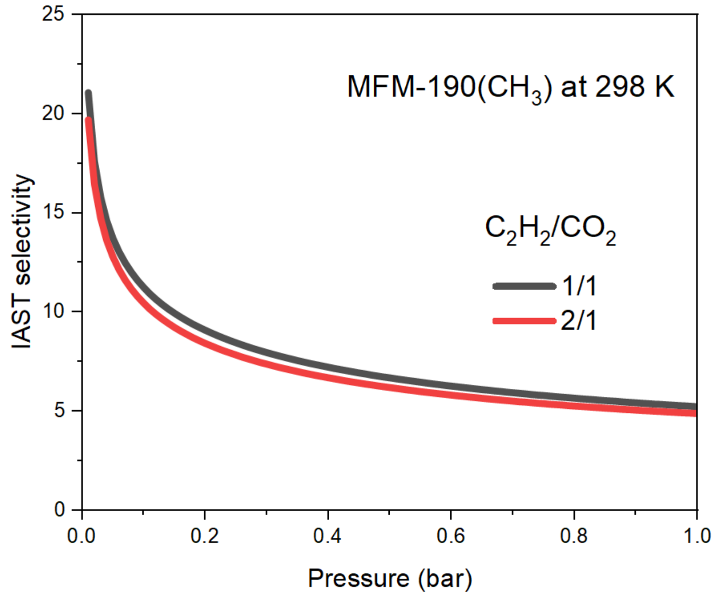

Figure S11. Analysis of IAST selectivities of C_2H_2/CO_2 (v/v = 1/1, 2/1) at 298 K in MFM-190(CH_3).

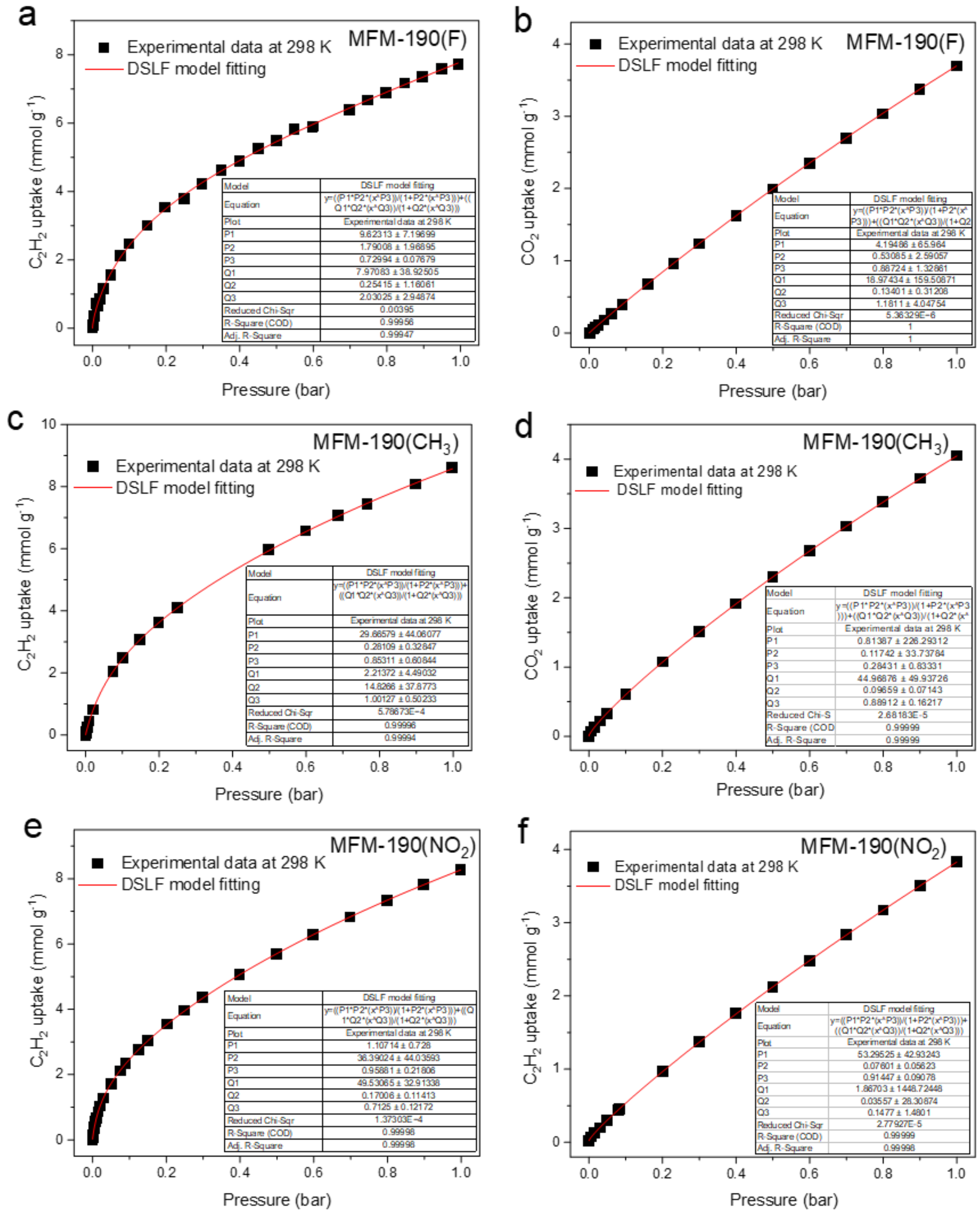

Figure S12. Fitting of C_2H_2 isotherms using the dual-site Langmuir Freundlich (DSLF) model for (a) MFM-190(F), (c) MFM-190(CH_3), (e) MFM-190(NO_2), and for CO_2 isotherms in (b) MFM-190(F), (d) MFM-190(CH_3), (f) MFM-190(NO_2) at 298 K.

6. Adsorption kinetics of C_2H_2 and CO_2

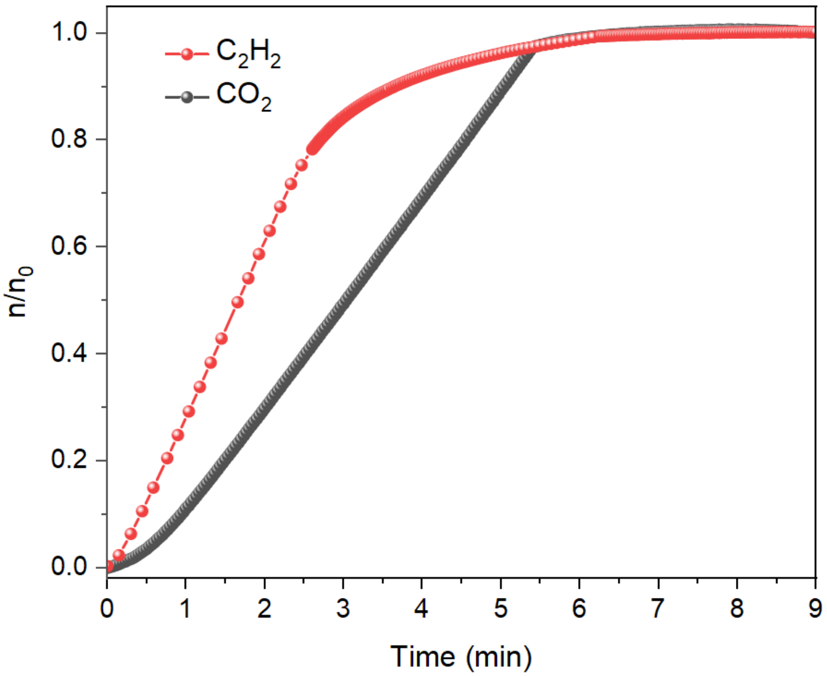

Figure S13. Adsorption kinetics of C_2H_2 and CO_2 in MFM-190(NO_2) at 298 K from 50 to 75 mbar.

7. Analysis and derivation of the isosteric heats of adsorption

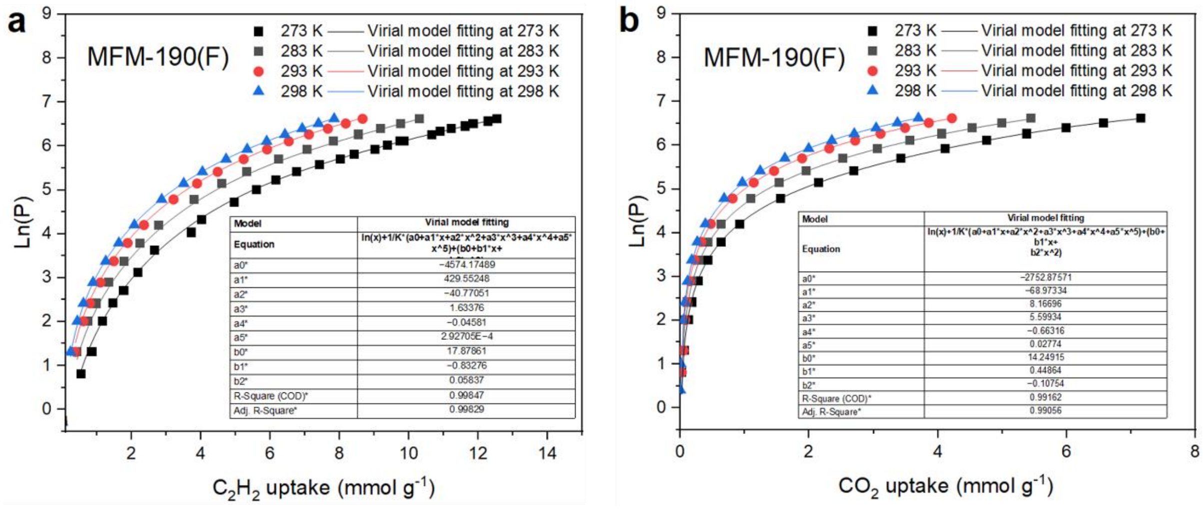

Figure S14. Virial model fitting of the (a) C_2H_2 and (b) CO_2 adsorption isotherms for MFM-190(F) at 273-298 K.

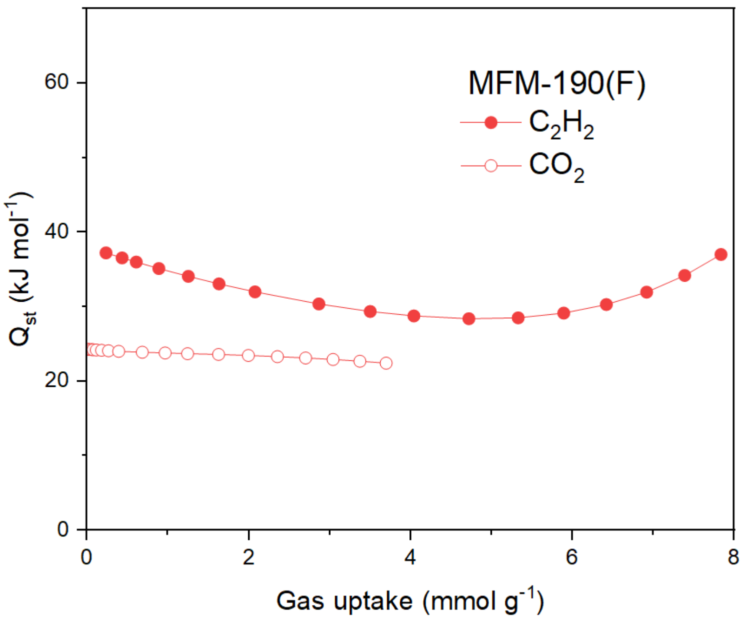

Figure S15. Isosteric heats of adsorption (Q_{st}) for C_2H_2 and CO_2 in MFM-190(F).

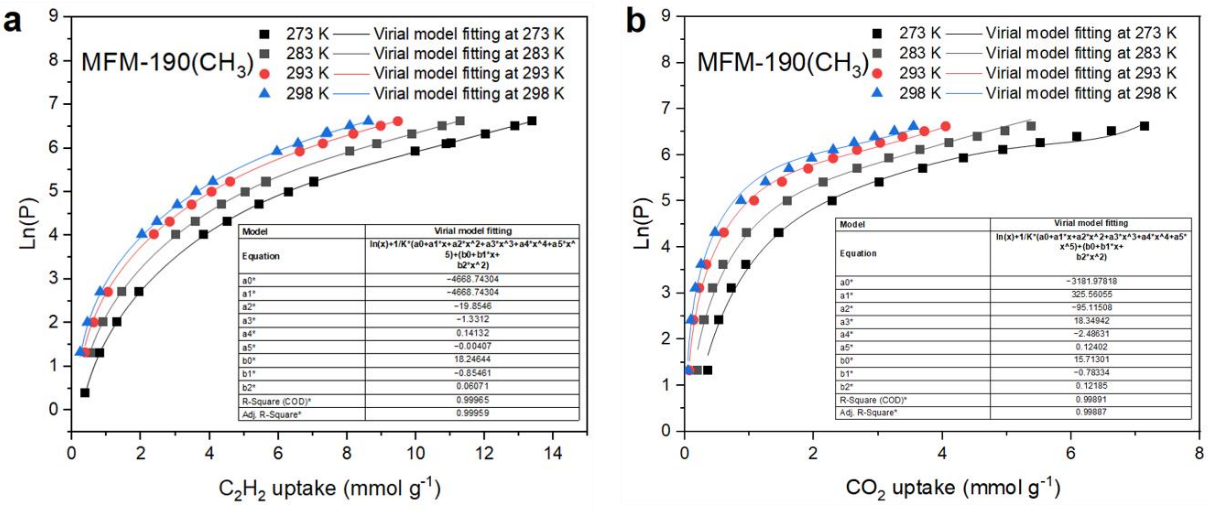

Figure S16. Virial model fitting of the (a) C_2H_2 and (b) CO_2 adsorption isotherms for MFM-190(CH_3) at 273 -298 K.

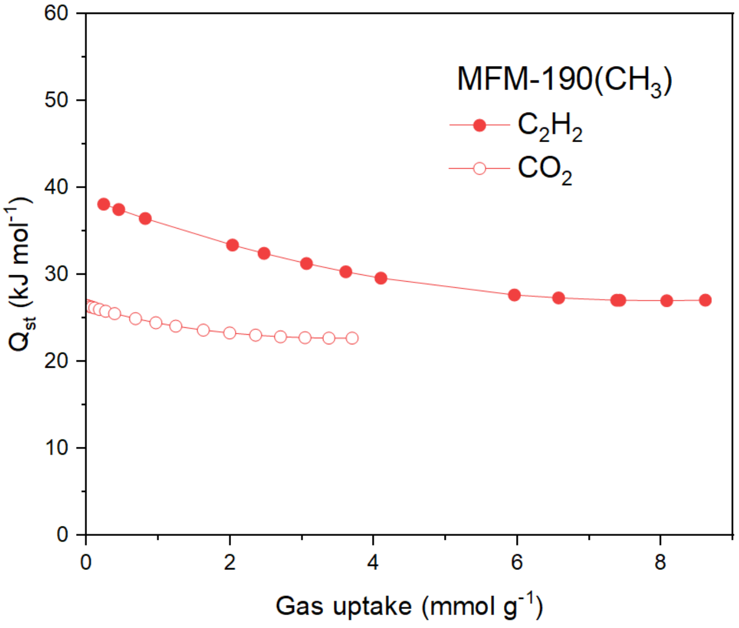

Figure S17. Isosteric heats of adsorption (Q_{st}) for C_2H_2 and CO_2 in MFM-190(CH_3).

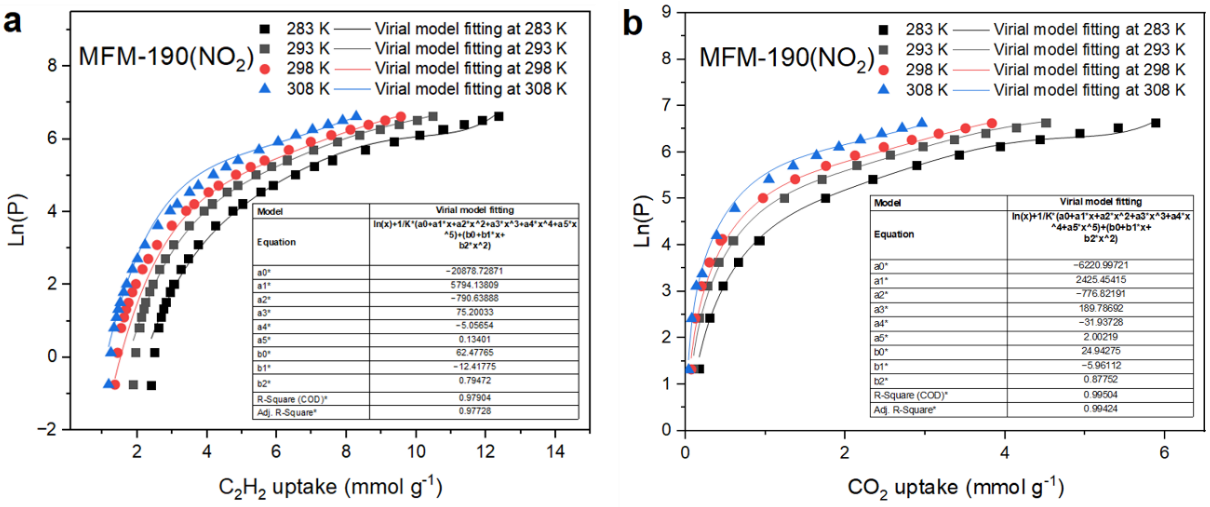

Figure S18. Virial model fitting of the (a) C_2H_2 and (b) CO_2 adsorption isotherms for MFM-190(NO_2) at 283 -308 K.

Due to the high affinity between C_2H_2 and MFM-190(NO_2), the value for Q_{st} was calculated using four isotherms collected at different temperatures (283–308 K) to improve accuracy.

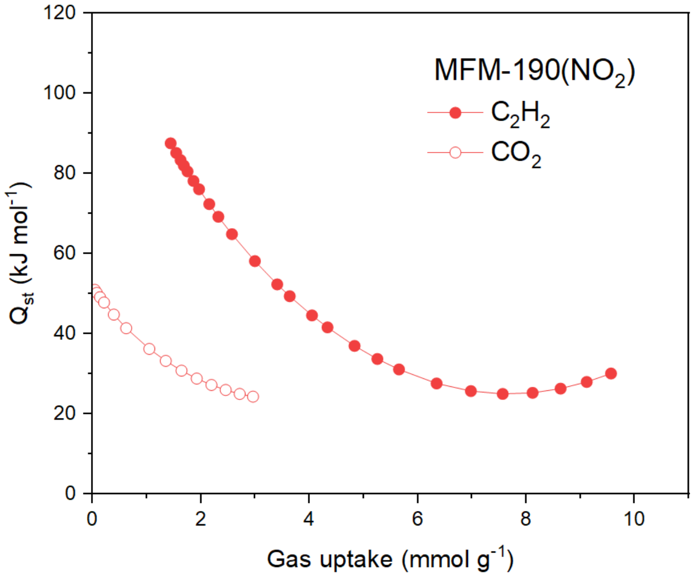

Figure S19. Isosteric heats of adsorption (Q_{st}) for C_2H_2 and CO_2 in MFM-190(NO_2).

8. Dynamic breakthrough experiments

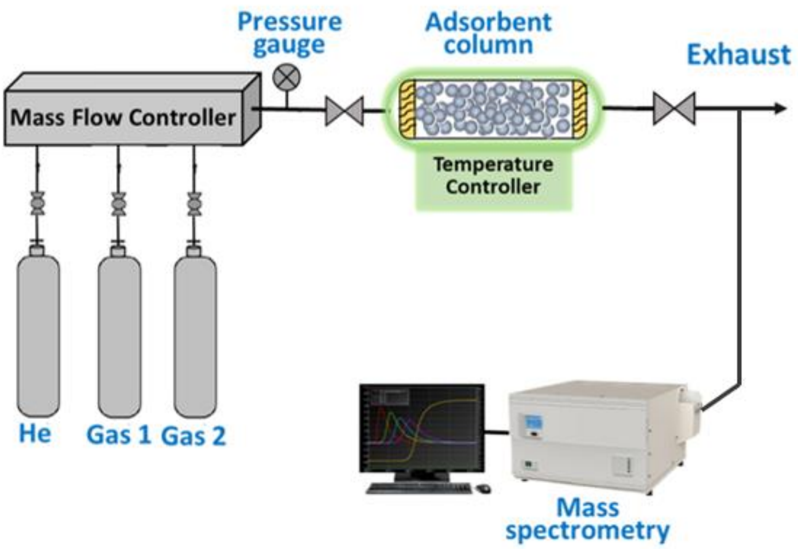

Figure S20. Illustration of apparatus for dynamic breakthrough experiments.

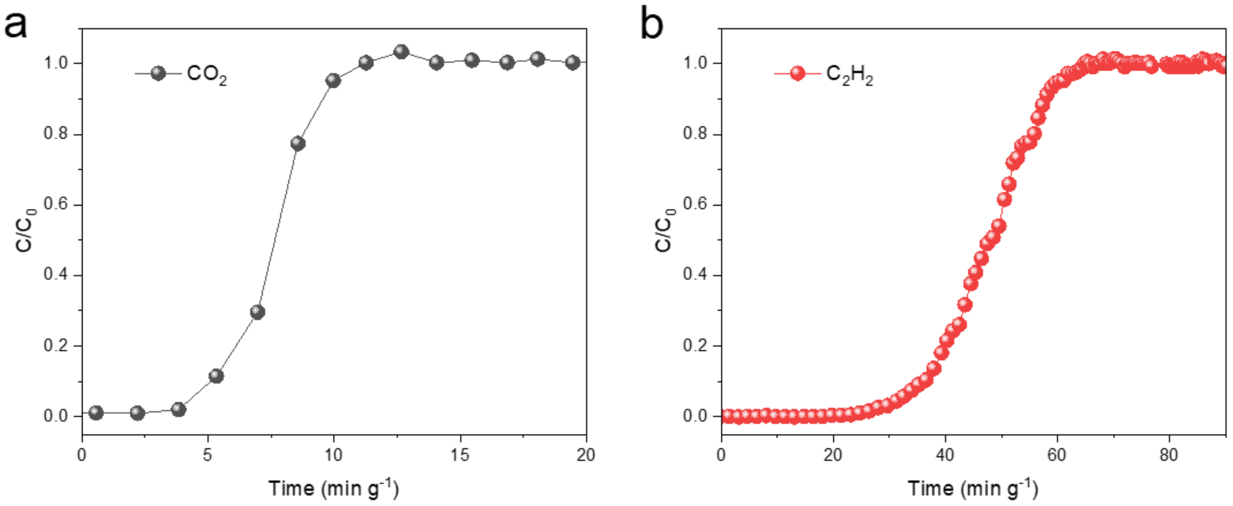

Figure S21. Column breakthrough curves of single components of (a) CO_2 and (b) C_2H_2 at 298 K and 1.0 bar under He flow (gas/He = 2 mL min^{-1}/18 mL min^{-1}, total gas flow: 20 mL min^{-1}; sample weight: 0.55 g) over a fixed-bed packed with MFM-190(NO_2).

9. Neutron powder diffraction

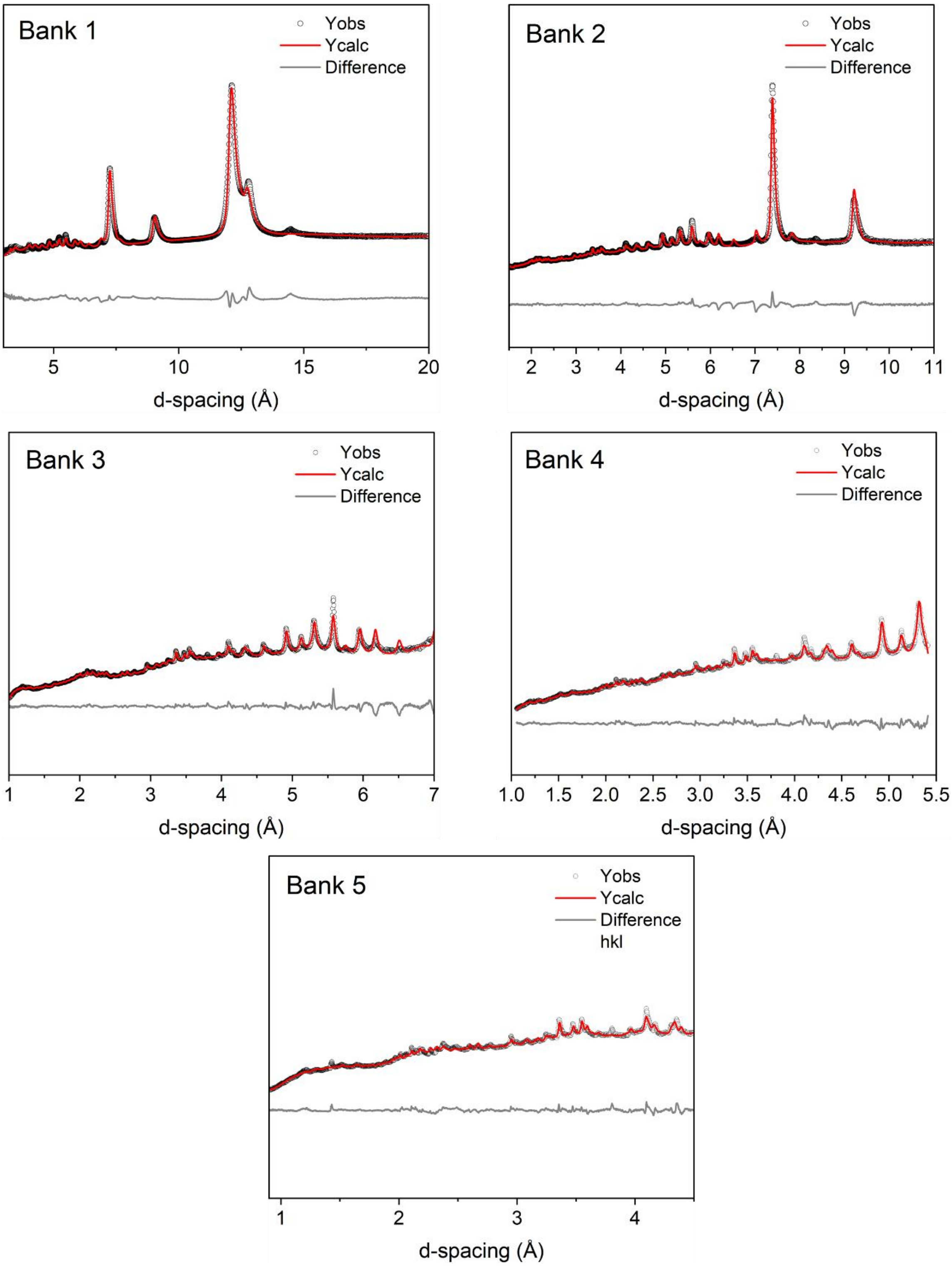

Figure S22. Rietveld refinement of NPD data for bare MFM-190(NO_2) from bank 1 to 5.

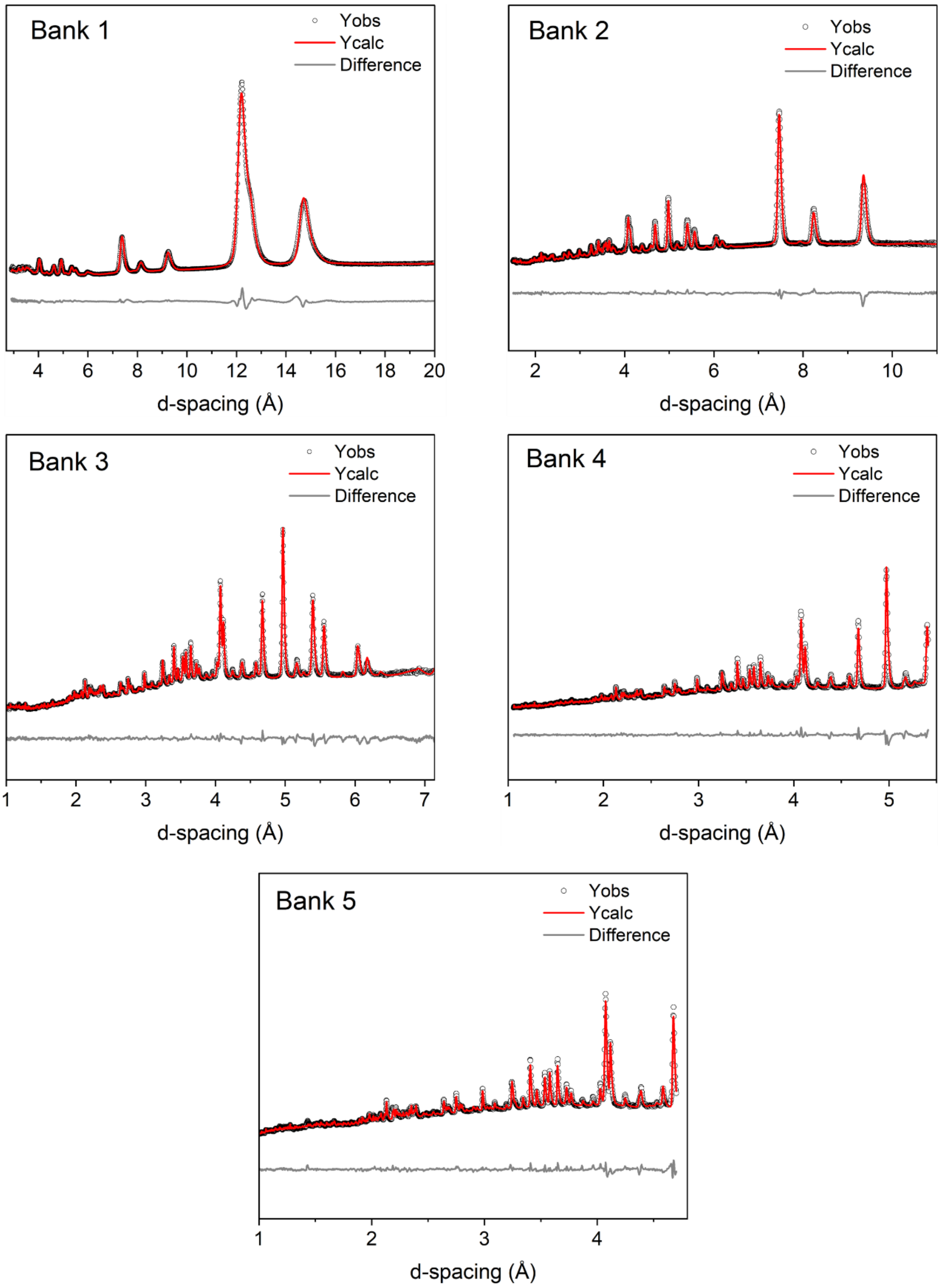

Figure S23. Rietveld refinement of NPD data for MFM-190(NO_2)·(C_2D_2)$_{5.2}$ from bank 1 to 5.

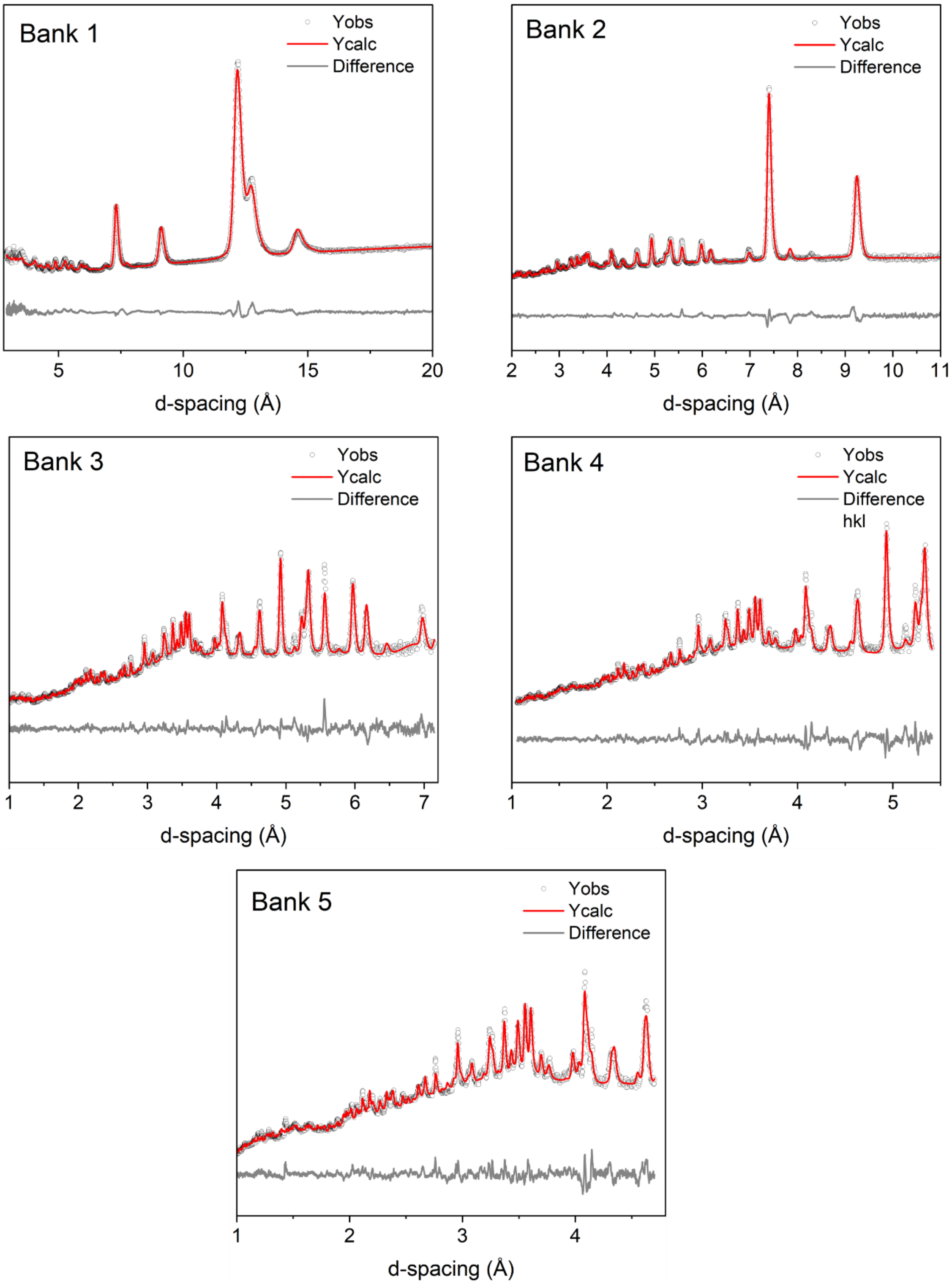

Figure S24. Rietveld refinement of NPD data for MFM-190(NO_2)·(CO_2)$_{3.1}$ from bank 1 to 5.

10. Supplementary tables

Table S1. Comparison of physical parameters for C_2H_2 and CO_2.

	C_2H_2	CO_2
Shape	linear	linear
Dimensions (Å)	3.3 × 3.3 × 5.7	3.2 × 3.3 × 5.4
Boiling points (K)	189.3	194.7
Critical temperature (K)	308.1	304.0
Critical pressure (bar)	62.7	73.8

Table S2. Comparison of C_2H_2 and CO_2 uptake capacities, C_2H_2/CO_2 uptake ratio, IAST selectivity, heats of adsorption (Q_{st}) and C_2H_2 productivity data of MFM-190(R) with the leading MOFs for C_2H_2/CO_2 separation in literature.

MOFs	Temperature (T)	Uptake ($cm^3\ g^{-1}$ STP) at 1bar		Uptake ratio (C_2H_2/CO_2)	IAST selectivity C_2H_2/CO_2=1/1	Q_{st} (kJ mol^{-1})		C_2H_2 producivity (mol kg^{-1})	Ref
		C_2H_2	CO_2			C_2H_2	CO_2		
MFM-190(NO_2)	298 K	215.8	86.4	2.5	~170–11<br>~150–11 (2/1)	75	33	4.0	This work
MFM-190(CH_3)	298 K	193.8	90.9	2.1	20–5.1	33	30	/	This work
MFM-190(F)	298 K	174.2	82.9	2.1	7.2–2.7	34	28	/	This work
MFM-190(H)/ZJU-5	298 K	193	89.1	2.17	/	35.8	/	/	[2, 3]
FJI-H8-Me	298 K	229	106	2.16	10.4–5.4	33.7	21.8	/	[4]
FJI-H8-Et	298 K	217	102	2.13		/	/	/	[4]
FJI-H8-^{n}Pr	298 K	179	92	1.94		/	/	/	[4]
FJI-H8-^{i}Pr	298 K	174	90	1.93		/	/	/	[4]
FJU-90a	298 K	180	103	1.75	6.4–4.3	25.2	20.7	4.16	[5]
SNNU-45	298 K	134	97.4	1.38	8.5–4.5	39.9	38.4	3.5	[6]
NOTT-101a	298 K	184	84	2.19	9–8	32.4	/	/	[7]
ZJU-40a	298 K	216	87	2.48	17–11.5	34.5	/	/	[8]
ZJU-50a	298 K	192	100	1.92	30–12	40	/	3.4	[9]
HKUST-1	298 K	198	137	1.44	2.4	34.8	/	/	[9]
SNNU-27-Fe	298 K	182.4	65.3	2.79	2	24.1	19.8	/	[10]
FJI-H36	298 K	160	100	1.6	3.7–3.5	36.1	29.2	3.82	[11]

FUT-1a	296 K	120	67.7	1.77	4	28.5	21.1	1.55	12
ATC-Cu	298 K	112	90	1.24	53.6	79	35	/	13
CAU-10	296 K	89.8	60.0	1.50	24.2–4.0	27	25	3.3	14
MIL-160(Al)	298 K	191	90	2.12	10	31.8	26.9	5.02	15
JNU-4a	298 K	222	150	1.48	8.2	26.8	19.7	/	16
Cu^{I}@UiO-66-$(COOH)_2$	298 K	48	22.4	2.14	185.0	74.5	28.9	2.89	17
JCM-1	298 K	75	38	1.97	13.7	36.9	33.4	2.2	18
UTSA-74a	298 K	108	90	1.61	22–9	31	25	/	19
BSF-3	298 K	80.4	47.3	1.70	16.3	42.7	22.4	2.44	20
BSF-3-Co	298 K	86.2	54.0	1.60	12.7	/	/	/	20
ZJU-74a	296 K	85.7	66.3	1.29	36.5	65	30	3.64	21
NKMOF-1-Ni	298 K	61.0	51.1	1.19	249.3–30	60.3	40.9	/	22
FJU-36a	296 K	52.2	35.5	1.47	2.8	32.9	31.1	0.46	23
FJU-89a	296 K	101.4	61.1	1.66	4.3–6.6	31.0	27.8	0.94	24
JXNU-12(F)	298 K	115.5	33.4	3.46	4.1	28.0	19.7	4.7	25
JXNU-12	298 K	77.9	37.3	2.08	2.0	21.3	19.9	3.1	25
SNNU-5-Sc	298 K	116.5	51.7	2.25	2.7	21.6	19.4	/	26
SNNU-150-Sc	298 K	47.1	25.4	1.85	8.2	25.8	22.1	/	26
ZJU-10a	298 K	174	81	2.15	9.7–4.0	39.0	25.0	/	27
SNNU-63	298 K	91.1	43.7	2.08	3.3	21.6	21.95	/	28

Table S3. Crystal data and structure refinements for bare MFM-190(NO_2), MFM-190(NO_2)·$(C_2D_2)_{5.2}$, and MFM-190(NO_2)·$(CO_2)_{3.1}$.

Compound	bare MFM-190(NO_2)	MFM-190(NO_2)·$(C_2D_2)_{5.2}$	MFM-190(NO_2)·$(CO_2)_{3.1}$
Empirical formula	$C_{21}Cu_2H_8N_2O_{10}$	$C_{31.4}Cu_2D_{10.4}H_8N_2O_{10}$	$C_{24.1}Cu_2H_8N_2O_{16.21}$
Formula weight	575.39	720.73	711.94
Crystal system	hexagonal	hexagonal	hexagonal
Space group	R-3m	R-3m	R-3m
a (Å)	18.3798(5)	18.6926(2)	18.4720(4)
b (Å)	18.3798(5)	38.2120(8)	18.4720(4)
c (Å)	39.002(3)	38.2120(8)	38.773(2)
α (deg)	90	90	90
β (deg)	90	90	90
γ (deg)	120	120	120
Volume ($Å^3$)	11410.2(11)	11563.0(3)	11457.4(8)
Z	9	9	9
ρ (calc) g/cm^3	0.7537	0.9316	0.9287
Method	Rietveld	Rietveld	Rietveld
Radiation type	Neutron	Neutron	Neutron
Scan method	Time of flight	Time of flight	Time of flight
R_{exp} (%)	0.31800	0.44929	0.91589
R_{wp} (%)	2.35516	1.98887	2.18312
R_p (%)	1.92172	1.60812	2.10988
GoF (χ^2)	7.40615	4.42664	2.38361
CCDC number	2310235	2310228	2310234

Table S4: Atomic positions for atoms in MFM-190(NO_2).

	x	y	z	Occupancy	Biso / Å^2
Cu1	1.0492(11)	0.5246(6)	0.5179(5)	1	2.0(6)
C1	0.9114(7)	0.5514(4)	0.5280(4)	1	3.0(2)
C2	0.8483(2)	0.5665(3)	0.5462(4)	1	3.0(2)
C3	0.8622(2)	0.5944(3)	0.5797(3)	1	3.0(2)
C4	0.80415(14)	0.6083(3)	0.5965(3)	1	3.0(2)
C5	0.77628(18)	0.5526(4)	0.5295(4)	1	3.0(2)
C6	0.81900(8)	0.63799(15)	0.63219(17)	1	3.0(2)
C7a	0.8927(4)	0.7143(3)	0.64084(13)	0.342(2)	3.0(2)
C7b	0.7596(4)	0.5904(4)	0.65801(6)	0.408(2)	3.0(2)
N7a	0.8927(4)	0.7143(3)	0.64084(13)	0.158(2)	3.0(15)
N7b	0.7596(4)	0.5904(4)	0.65801(6)	0.092(2)	3.0(15)
H7a	0.9358(9)	0.7488(8)	0.6221(4)	0.25	3.6(3)
H7b	0.7061(11)	0.5350(11)	0.65174(14)	0.25	3.6(3)
O1	0.9785(10)	0.5651(6)	0.5445(6)	1	0.5(3)
O2	0.8964(14)	0.5251(5)	0.4965(5)	1	0.5(3)
N8a	0.954(2)	0.7636(18)	0.6141(9)	0.092(2)	3.0(15)
O3a	1.034(4)	0.803(4)	0.620(2)	0.092(2)	0.5(3)
O4a	0.930(5)	0.770(4)	0.5834(16)	0.092(2)	0.5(3)
N8b	0.683(3)	0.511(3)	0.6491(3)	0.158(2)	3.0(15)
O3b	0.685(5)	0.461(5)	0.6262(13)	0.158(2)	0.5(3)
O4b	0.612(4)	0.490(5)	0.6638(15)	0.158(2)	0.5(3)

Table S5. Host–guest interactions in MFM-190(NO_2)·(C_2D_2)$_{5.2}$.

MFM-190(NO_2)·(C_2D_2)$_{5.2}$	Interactions	Distance (Å)	Colour
Site I	C≡C (site I)···Cu	3.05(2)	yellow
	C≡C (site I)···C≡C (site II)	3.39(1)	purple
	C≡C (site I)···C≡C (site IV)	2.55(1)	green
Site II	C≡C (site II)···C≡C (site I)	3.39(1)	purple
	D (site II)···phenyl groups	2.81(3)	dark purple
	D (site II)···O_{NO2}	2.54(1)	dark blue
Site III	D (site III)···Cu	2.75(4)	dark orange
	D (site III)···O_{ligand}	2.67(1)	sea cyan
Site IV	C≡C (site IV)···Cu	3.35(1)	green
Site V	C≡C (site V)···C≡C (site VI)	2.68(2)	pink
Site VI	C≡C (site VI)···pyridyl sites	2.99(1)	violet
	C≡C (site V)···C≡C (site VI)	2.68(2)	pink
Site VII	C≡C (site VII)···phenyl groups	3.96(1)	balck
	C≡C (site VII)···phenyl groups	3.79(2)	black

Table S6: Atomic positions for atoms in MFM-190(NO_2)·(C_2D_2)$_{5.2}$.

	x	y	z	Occupancy	Biso / Å^2
Cu1	1.0438(4)	0.5219(2)	0.5223(2)	1	2.0(2)
C1_1	0.8588(3)	0.3847(9)	0.41976(17)	0.359(3)	4.0(4)
C2_1	0.8621(3)	0.3221(9)	0.42092(17)	0.359(3)	4.0(4)
D1_1	0.8558(3)	0.4398(9)	0.41874(17)	0.359(3)	4.0(4)
D2_1	0.8650(3)	0.2671(9)	0.42194(17)	0.359(3)	4.0(4)
C1_2	0.6910(12)	0.2944(8)	0.2012(2)	0.185(2)	1.0(5)
C2_2	0.6402(12)	0.3136(8)	0.1940(2)	0.185(2)	1.0(5)
D1_2	0.7356(12)	0.2775(8)	0.2076(2)	0.185(2)	1.0(5)
D2_2	0.5955(12)	0.3305(8)	0.1876(2)	0.185(2)	1.0(5)
C1_3	0.1361(12)	0.8567(18)	0.9389(4)	0.180(3)	4.0(7)
C2_3	0.1543(12)	0.8049(18)	0.9458(4)	0.180(3)	4.0(7)
D1_3	0.1202(12)	0.9022(18)	0.9329(4)	0.180(3)	4.0(7)
D2_3	0.1702(12)	0.7594(18)	0.9518(4)	0.180(3)	4.0(7)
C1_4	0.6594(7)	0.8791(6)	0.1185(7)	0.192(3)	4.0(8)
C2_4	0.6604(7)	0.8890(6)	0.1496(7)	0.192(3)	4.0(8)
D1_4	0.6585(7)	0.8704(6)	0.0911(7)	0.192(3)	4.0(8)
D2_4	0.6613(7)	0.8977(6)	0.1770(7)	0.192(3)	4.0(8)
C1_5	0.643(15)	0.354(16)	0.5862(11)	0.076(2)	1.0(12)
C2_5	0.637(15)	0.364(16)	0.6169(11)	0.076(2)	1.0(12)
D1_5	0.649(15)	0.345(16)	0.5592(11)	0.076(2)	1.0(12)
D2_5	0.631(15)	0.373(16)	0.6439(11)	0.076(2)	1.0(12)
C1_6	0.3694(10)	0.4829(8)	0.2732(6)	0.168(4)	4.0(10)
C2_6	0.4156(10)	0.4778(8)	0.2937(6)	0.168(4)	4.0(10)
D1_6	0.3288(10)	0.4874(8)	0.2552(6)	0.168(4)	4.0(10)
D2_6	0.4562(10)	0.4733(8)	0.3117(6)	0.168(4)	4.0(10)
C1_7	0.0983(13)	0.0333(19)	0.6730(5)	0.135(3)	3.8(10)
C2_7	0.0991(13)	0.0973(19)	0.6738(5)	0.135(3)	3.8(10)
D1_7	0.0975(13)	0.0229(19)	0.6724(5)	0.135(3)	3.8(10)
D2_7	0.0998(13)	0.1535(19)	0.6744(5)	0.135(3)	3.8(10)
C1	0.9065(3)	0.5455(2)	0.5269(2)	1	3.00(8)
C2	0.84472(11)	0.56157(19)	0.54536(18)	1	3.00(8)
C3	0.85941(12)	0.59094(16)	0.57926(17)	1	3.00(8)
C4	0.80281(8)	0.60563(15)	0.59621(17)	1	3.00(8)

C5	0.77344(10)	0.5469(2)	0.52841(19)	1	3.00(8)
C6	0.81848(4)	0.63695(8)	0.63237(9)	1	3.00(8)
C7a	0.89919(18)	0.6905(2)	0.64349(7)	0.337(2)	3.00(8)
C7b	0.7526(2)	0.6131(3)	0.65554(5)	0.413(2)	3.00(8)
O1	0.9732(4)	0.5598(3)	0.5433(3)	1	3.00(18)
O2	0.8914(6)	0.5179(2)	0.4949(2)	1	3.00(18)
O3a	1.038(2)	0.7227(19)	0.6295(11)	0.087(2)	3.00(18)
O3b	0.651(3)	0.495(2)	0.6233(8)	0.163(2)	3.00(18)
O4a	0.962(3)	0.7315(18)	0.5865(9)	0.087(2)	3.00(18)
O4b	0.607(3)	0.567(3)	0.6537(9)	0.163(2)	3.00(18)
N7a	0.89919(18)	0.6905(2)	0.64349(7)	0.163(2)	3.0(6)
N7b	0.7526(2)	0.6131(3)	0.65554(5)	0.087(2)	3.0(6)
N8a	0.9685(11)	0.7157(6)	0.6191(4)	0.087(2)	3.0(6)
N8b	0.6676(13)	0.5567(10)	0.6438(2)	0.163(2)	3.0(6)
H5	0.76248(14)	0.5250(3)	0.5031(3)	1	3.60(10)
H3	0.9126(4)	0.60190(18)	0.59191(19)	1	3.60(10)
H7a	0.9478(4)	0.7082(4)	0.62640(16)	0.25	3.60(10)
H7b	0.6931(5)	0.5735(5)	0.64733(9)	0.25	3.60(10)

Table S7. Host–guest interactions in MFM-190(NO_2)·(CO_2)$_{3.1}$.

MFM-190(NO_2)·(CO_2)$_{3.1}$	Interactions	Distances (Å)	Colour
Site I	C=O (site I)···phenyl groups	3.94(1)	yellow
Site II	C=O (site II)···$H_{phenyl\ groups}$	2.99(1)	pink
	C=O (site II)···Cu	3.32(1)	purple
	C=O (site II)···C=O (site III)	3.57(7)	orange
Site III	C=O (site III)···pyridyl sites	3.68(4)	orange
Site IV	C=O (site IV)···phenyl rings	3.99(2)	green
	C=O (site IV)···Cu	4.37(1)	light green

Table S8: Atomic positions for atoms in MFM-190(NO_2)·$(CO_2)_{3.1}$.

	x	y	z	Occupancy	Biso / Å^2
Cu1	1.0470(7)	0.5235(3)	0.5220(3)	1	1.5(4)
C1_1	0.6398(5)	0.4976(7)	0.4089(3)	0.378(4)	4.0(6)
O1_1	0.6217(11)	0.4328(8)	0.4205(5)	0.378(4)	4.0(6)
O2_1	0.6578(11)	0.5624(8)	0.3973(5)	0.378(4)	4.0(6)
C1_2	0	0	0.7760(11)	0.394(10)	3.8(14)
O1_2	0	0	0.7461(11)	0.394(10)	3.8(14)
O2_2	0	0	0.8059(11)	0.394(10)	3.8(14)
C1_3	0.133(4)	0.891(4)	0.6921(4)	0.167(4)	1.6(16)
O1_3	0.162(6)	0.849(6)	0.6873(8)	0.167(4)	1.6(16)
O2_3	0.103(6)	0.933(6)	0.6968(8)	0.167(4)	1.6(16)
C1_4	0.2169(10)	0.9013(14)	0.5270(6)	0.165(3)	1.0(12)
O1_4	0.2114(18)	0.8410(16)	0.5388(10)	0.165(3)	1.0(12)
O2_4	0.2223(18)	0.9615(16)	0.5152(10)	0.165(3)	1.0(12)
C1	0.9095(5)	0.5490(3)	0.5268(3)	1	3.00(12)
C2	0.84689(15)	0.5644(3)	0.5451(2)	1	3.00(12)
C3	0.86103(16)	0.5927(2)	0.5787(2)	1	3.00(12)
C4	0.80341(10)	0.6068(2)	0.5955(2)	1	3.00(12)
C5	0.77512(14)	0.5502(3)	0.5283(3)	1	3.00(12)
C6	0.81878(5)	0.63756(11)	0.63207(12)	1	3.00(12)
C7a	0.8977(2)	0.7058(3)	0.64168(9)	0.470(3)	3.00(12)
C7b	0.7544(3)	0.5984(3)	0.65705(5)	0.280(3)	3.00(12)
O1	0.9750(7)	0.5624(4)	0.5427(4)	1	3.0(3)
O2	0.8953(9)	0.5231(3)	0.4960(3)	1	3.0(3)
H3	0.9146(6)	0.6032(2)	0.5913(3)	1	3.60(15)
H5	0.76457(19)	0.5291(4)	0.5032(4)	1	3.60(15)
N7a	0.8977(2)	0.7058(3)	0.64168(9)	0.030(3)	3.0(8)
N7b	0.7544(3)	0.5984(3)	0.65705(5)	0.220(3)	3.0(8)
H7a	0.8977(2)	0.7058(3)	0.64168(9)	0.030(3)	3.0(8)
H7b	0.7544(3)	0.5984(3)	0.65705(5)	0.220(3)	3.0(8)
N8a	0.9644(15)	0.7463(10)	0.6158(6)	0.220(3)	3.0(8)
O3a	0.955(3)	0.717(3)	0.5846(11)	0.220(3)	3.0(3)
O4a	1.035(3)	0.813(2)	0.6234(13)	0.220(3)	3.0(3)
N8b	0.6726(19)	0.5278(17)	0.6471(2)	0.030(3)	3.0(8)
O3b	0.615(3)	0.487(3)	0.6700(11)	0.030(3)	3.0(3)

O4b	0.656(3)	0.504(3)	0.6151(9)	0.030(3)	3.0(3)

11. Supplementary references

(1) W. Y. Li, J. N. Li, T. D. Duong, S. A. Sapchenko, X. Han, J. D. Humby, G. F. S. Whitehead, I. J. V. Yrezábal, I. Silva, P. Manuel, M. D. Frogley, G. Cinque, M. Schröder, S. H. Yang, *J. Am. Chem. Soc.* **2022**, *144*, 13196–13204.

(2) X. T. Rao, J. F. Cai, J. C. Yu, Y. B. He, C. D. Wu, W. Zhou, T. Yildirim, B. L. Chen, G. D. Qian, *Chem. Commun.* **2013**, *49*, 6719–6721.

(3) M. H. He, T. T. Xu, Z. Z. Jiang, L. Y. Yang, Y. Zhou, F. J. Xia, X. Wang, X. J. Wang, Y. B. He, *Inorg. Chem. Front.* **2019**, *6*, 1177–1183.

(4) Z. Y. Di, C. P. Liu, J. D. Pang, C. Chen, F. L. Hu, D. Q. Yuan, M. Y. Wu, M. C. Hong, *Angew. Chem., Int. Ed.* **2021**, *60*, 10828–10832.

(5) Y. X. Ye, Z. L. Ma, R. B. Lin, R. Krishna, W. Zhou, Q. J. Lin, Z. J. Zhang, S. C. Xiang, B. L. Chen, *J. Am. Chem. Soc.* **2019**, *141*, 4130–4136.

(6) Y. P. Li, Y. Wang, Y. Y. Xue, H. P. Li, Q. G. Zhai, S. N. Li, Y. C. Jiang, M. C. Hu, X. H. Bu, *Angew. Chem., Int. Ed.* **2019**, *131*, 13724–13729.

(7) Y. B. He, R. Krishna, B. L. Chen, *Energy Environ. Sci.* **2012**, *5*, 9107–9120.

(8) H. M. Wen, H. Z. Wang, B. Li, Y. J. Cui, H. L. Wang, G. D. Qian, B. L. Chen, *Inorg. Chem.* **2016**, *55*, 7214–7218.

(9) K. Shao, H. M. Wen, C. C. Liang, X. Y. Xiao, X. W. Gu, B. L. Chen, G. D. Qian, B. Li, *Angew. Chem., Int. Ed.* **2022**, e202211523.

(10) Y. Y. Xue, X. Y. Bai, J. Zhang, Y. Wang, S. N. Li, Y. C. Jiang, M. C. Hu, Q. G. Zhai, *Angew. Chem., Int. Ed.* **2021**, *60*, 10122–10128.

(11) J. D. Tian, Q. H. Chen, F. L. Jiang, D. Q. Yuan, M. C. Hong, *Angew. Chem., Int. Ed.* **2023**, *62*, e202215253.

(12) L. Z. Liu, S. S. Wu, D. D. Li, Y. B. Li, H. Zhang, L. Li, S. W. Jin, Z. Z. Yao, *ACS Appl. Mater. Inter.* **2022**, *14*, 36882–36889.

(13) Z. Niu, X. L. Cui, T. Pham, G. Verma, P. C. Lan, C. Shan, H. B. Xing, K. A. Forrest, S. Suepaul, B. Space, A. Nafady, A. M. Enizi, S. Q. Ma, *Angew. Chem., Int. Ed.* **2021**, *60*, 5283–5288.

(14) J. Y. Pei, H. M. Wen, X. W. Gu, Q. L. Qian, Y. Yang, Y. J. Cui, B. Li, B. L. Chen, G. D. Qian, *Angew. Chem., Int. Ed.* **2021**, *60*, 25068–25074.

(15) Y. X. Ye, S. K. Xian, H. Cui, K. Tan, L. S. Gong, B. Liang, T. Pham, H. Pandey, R. Krishna, P. C. Lan, K. A. Forrest, B. Space, T. Thonhauser, J. Li, S. Q. Ma, *J. Am. Chem. Soc.* **2022**, *144*, 1681–1689.

(16) H. Zeng, X. J. Xie, Y. Wang, D. Luo, R. J. Wei, W. G. Lu, D. Li, *Chem. Sci.* **2022**, *13*, 12876–12882.

(17) L. Zhang, K. Jiang, L. F. Yang, L. B. Li, E. Hu, L. Yang, K. Shao, H. B. Xing, Y. J. Cui, Y. Yang, B. Li, B. L. Chen, G. D. Qian, *Angew. Chem., Int. Ed.* **2021**, *60*, 15995–16002.

(18) J. Lee, C. Y. Chuah, J. Kim, Y. Kim, N. Ko, Y. Seo, K. Kim, T. H. Bae, E. Lee, *Angew. Chem., Int. Ed.* **2018**, *130*, 7995–7999.

(19) F. Luo, C. S. Yan, L. L. Dang, R. Krishna, W. Zhou, H. Wu, X. L. Dong, Y. Han, T. L. Hu, M. O'Keeffe, L. L. Wang, M. B. Luo, R. B. Lin, B. L. Chen, *J. Am. Chem. Soc.* **2016**, *138*, 5678–5684.

(20) Y. B. Zhang, J. B. Hu, R. Krishna, L. Y. Wang, L. F. Yang, X. L. Cui, S. Duttwyler, H. B. Xing, *Angew. Chem., Int. Ed.* **2020**, *59*, 17664–17669.

(21) J. Y. Pei, K. Shao, J. X. Wang, H. M. Wen, Y. Yang, Y. J. Cui, R. Krishna, B. Li, G. D. Qian, *Adv. Mater.* **2020**, *32*, 1908275.

(22) Y. L. Peng, T. Pham, P. F. Li, T. Wang, Y. Chen, K. J. Chen, K. A. Forrest, B. Space, P. Cheng, M. J. Zaworotko, *Angew. Chem., Int. Ed.* **2018**, *57*, 10971–10975.

(23) L. Z. Liu, Z. Z. Yao, Y. X. Ye, L. J. Chen, Q. J. Lin, Y. S. Yang, Z. J. Zhang, S. C. Xiang, *Inorg. Chem.* **2018**, *57*, 12961–12968.

(24) Y. X. Ye, S. M. Chen, L. J. Chen, J. T. Huang, Z. L. Ma, Z. Y. Li, Z. Z. Yao, J. D. Zhang, Z. J. Zhang, S. C. Xiang, *ACS Appl. Mater. Inter.* **2018**, *10*, 30912–30918.

(25) X. P. Fu, Y. L. Wang, X. F. Zhang, R. Krishna, C. T. He, Q. Y. Liu, B. L. Chen, *Chem. Eng. J.* **2022**, *432*, 134433.

(26) H. J. Lv, J. W. Zhang, Y. C. Jiang, S. N. Li, M. C. Hu, Q. G. Zhai, *Inorg. Chem.* **2022**, *61*, 3553–3562.

(27) X. Duan, H. Z. Wang, Z. G. Ji, Y. J. Cui, Y. Yang, G. D. Qian, *J. Solid State Chem.* **2016**, *241*, 152–156.

(28) Y. T. Li, J. W. Zhang, H. J. Lv, M. C. Hu, S. N. Li, Y. C. Jiang, Q. G. Zhai, *Inorg. Chem.* **2020**, *59*, 10368–10373.
